# Generating repairs for inconsistent models

**DOI:** 10.1007/s10270-022-00996-0

**Published:** 2022-04-04

**Authors:** Luciano Marchezan, Roland Kretschmer, Wesley K. G. Assunção, Alexander Reder, Alexander Egyed

**Affiliations:** 1 Institute for Software Systems Engineering, Johannes Kepler University, Linz, Austria; 2 Dynatrace Research, Linz, Austria; 3 MIC Datenverarbeitung, Linz, Austria

**Keywords:** Model-driven engineering, Inconsistency repair, Consistency checking, Repair generation

## Abstract

There are many repair alternatives for resolving model inconsistencies, each involving one or more model changes. Enumerating them all could overwhelm the developer because the number of possible repairs can grow exponentially. To address this problem, this paper focuses on the immediate cause of an inconsistency. By focusing on the cause, we can generate a repair tree with a subset of repair actions focusing on fixing this cause. This strategy identifies model elements that must be repaired, as opposed to additional model elements that may or may not have to be repaired later. Furthermore, our approach can provide an ownership-based filter for filtering repairs that modify model elements not owned by a developer. This filtering can further reduce the repair possibilities, aiding the developer when choosing repairs to be performed. We evaluated our approach on 24 UML models and four Java systems, using 17 UML consistency rules and 14 Java consistency rules. The evaluation data contained 39,683 inconsistencies, showing our approach’s usability as the repair trees sizes ranged from five to nine on average per model. Also, these repair trees were generated in 0.3 seconds on average, showing our approach’s scalability. Based on the results, we discuss the correctness and minimalism with regard to the cause of the inconsistency. Lastly, we evaluated the filtering mechanism, showing that it is possible to further reduce the number of repairs generated by focusing on ownership.

## Introduction

State of the art on inconsistency management in model-based software development has focused on detecting inconsistencies. Today, many approaches are available to detect inconsistencies quickly and correctly [6, 12, 17, 32]. While it is important to tolerate inconsistencies [4], they must be resolved eventually. Unfortunately, repairing inconsistencies is much harder than detecting them because the number of alternatives grows exponentially with the number of model elements accessed [42].

Existing approaches either ignore this exponential growths [33] or emphasize on selected repairs only [11, 40]. Other approaches, however, pose limitations on the consistency language or focus on individual inconsistencies [14, 33, 54]. All these limitations are problematic because developers must choose repair alternatives from a large set of possibilities. This selection can either overwhelm the developer or may fail to include the repairs the developer desires. Since the repairing of inconsistencies goes hand in hand with the creative process of modeling, we strongly advocate against heuristics that replace the role of the (human) developer. For example, a repair that identifies the least number of model changes may be undesirable as it may, for example, favor the undoing of a change that caused the inconsistency [36].

Another aspect not addressed in the literature is the process of repairing inconsistencies in a collaborative environment. As software engineering is mostly a team-based discipline [31], repairing engineering models can also be done in collaboration. Collaboration aspects can be used for dealing with the large set of repair alternatives, filtering out non-feasible options. For instance, when deciding which repairs to be executed, an engineer may want to focus on the repairs that impact only the artifacts which are related to him/her. In this sense, an ownership property from the artifacts being modified by repair alternatives can be used for reducing these possibilities. However, there are no repair approaches considering collaborative environments reported in the literature [29, 49].

Based on the limitations existing in the literature and to deal with practical needs of developers, the main contribution of this paper is a scalable approach for generating repair alternatives focusing on the cause of inconsistencies. Furthermore, in this paper, we extend past work [44] by considering the ownership of model elements. This ownership is used for highlighting repair actions based on the model elements being modified. Then, a filtering mechanism is applied for generating a set of possible repairs from the repair tree based on the highlighting. This filtering can be used for reducing the amount of alternatives, addressing the problem related with exponential growth of repair alternatives. Despite being a generic approach, in this paper we also present as a technical contribution an implementation of our approach as the DesignSpace-IR^1^ tool, which is part of the DesignSpace project.^2^ The main reason for implementing a new tool is to extend the Model/Analyzer tool [42], by giving support for consistency checking and repair generation for other models, besides UML, which is the only model supported by the Model/Analyzer.

Inconsistencies can be repaired even if the repair extends beyond the immediate cause, which is commonly the case [12]. Our approach combines knowledge about the structure of inconsistent consistency rules, as in xLinkit [32], with the consistency rules’ expected and observed results, as in Egyed [13]. Basic facts about the meta-model, such as types of fields and non-changeable model elements, are used to remove inapplicable repairs. The structure of consistency rules is important for enumerating repair alternatives conservatively.

For example, if a consistency rule $a \wedge b$ is inconsistent, *i.e.*, *false*, then there are three repair alternatives as generated by xLinkit: repair *a*, repair *b*, or repair *a* and *b*. This list appears reasonable, but it may contain unneeded repair alternatives. Let us suppose that merely *a* was *false* and *b* was *true*, then (i) repair *b* would be incorrect because it would fail to repair *a*, which is broken; and (ii) repair *a* and *b* is non-minimal because it would repair more than immediately necessary. By focusing on the cause, which is *a* in this case, our approach provides a subset of repair actions focusing on repairing *a*, rather than *a* and *b*. However, the repair of *a* may inadvertently break *b* or another consistency rule as a side effect. This potential side effect depends on the course of action chosen by the developer. Despite the need of repairing *a*, while avoiding to break *b*, any side effect can only be observed after applying the repair for *a*.

By focusing on the cause of an inconsistency, our approach focuses on a smaller, more manageable problem *that must be repaired*. Hence, we do not focus on the much larger problem of potential side effects that *may or may not need to be repaired* later. Thus, we argue that our approach is minimal with regard to the cause of an inconsistency. Moreover, the repair actions provided by our approach represent a subset of repair alternatives. From this subset, one repair alternative must be performed for repairing the cause of the inconsistency.

Our approach builds on xLinkit and removes repair alternatives that do not repair the cause of a given inconsistency. In doing so, our approach is still able to retain a complete set of repair alternatives while not necessarily identifying all possible repair actions within. Yet, the literature distinguishes abstract and concrete repair actions [32] where abstract actions identify what model elements to change, *e.g.*, change element *A*, and concrete actions identify how to change them, *e.g.*, change element *A* to *true*. Our approach identifies abstract repair actions though it is also possible to compute concrete repair actions in many situations. Additionally, since there may exist multiple alternatives for repairing inconsistencies and these alternatives may overlap in repair actions, our approach organizes repair alternatives/actions in a hierarchical repair tree. This tree structures the way repair actions may be selected and developers may find it more useful as a decision-making tool.

Our approach’s usability and scalability were empirically evaluated on 24 UML and four Java systems in the context of 39,683 inconsistencies. The results showed an average size of repair trees ranging from five to nine repair actions per model. This evidence our approach usability as such range is reasonable for the developer to deal with when selecting repairs. Scalability results showed that the generation of evaluation trees averaged 4.69 seconds, while the generation of repair trees takes 0.3 seconds on average. Correctness and minimalism are argued in comparison to Nentwich et al. [32, 33], showing that our approach is minimal with regard to the cause. Furthermore, we evaluated the ownership filtering mechanism considering user-based and artifact-based ownership. The results show that the filter reduces the number of repairs generated based on the number of model elements owned. Hence, if, for instance, 50% of the model elements are owned, the number of repairs is reduced to 50% of the total amount approximately.

The paper is organized as follows. Section 2 illustrates the problem of generating repairs and provides basic definitions. Section 3 defines the goals of this paper and how it extends the conference version that precedes this paper [42]. Section 4 discusses the principles of our approach. Section 5 shows the evaluation of our approach which is followed by Sect. 6 on the threats to validity. Section 7 gives an overview of the related work on this topic, and finally, Sect. 8 concludes the paper and gives an outlook on future work.

## Problem and definitions

**Fig. 1:**
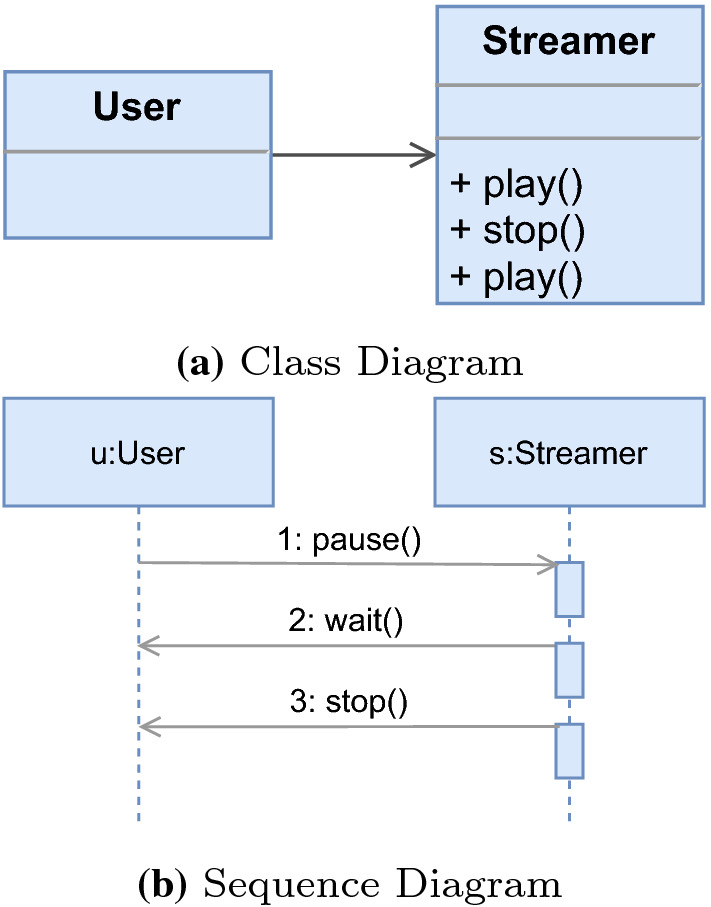
UML Model snippets of a VOD System

To illustrate the problem, we introduce an excerpt of a video on demand (VOD) system based on a client–server architecture and a set of consistency rules that specify well-formedness. For illustrative purposes, we use a simplified version of UML [38] and Object Constraint Language (OCL) [37]. The model shown in Fig. 1 consists of two diagrams: Fig. 1a) a class diagram describing the structure of the VOD system, and Fig. 1b) a sequence diagram describing the process of pausing, waiting, and stopping a video.

Inconsistencies arise if the models violate basic well-formedness constraints expressed as consistency rules (CRs). We use two such rules for illustration. Firstly, we present CR 1 that checks if the operation names in a class are unique. More specifically, an inconsistency is detected if there are two different operations (*o1*$<>$*o2*) with the same name (*o1.name* $=$ *o2.name*) in the same class. In the model of our example, this CR is violated by the class *Streamer* (Fig. 1a) because two operations with the same name, *play*, exist.
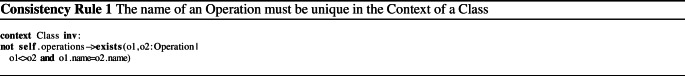

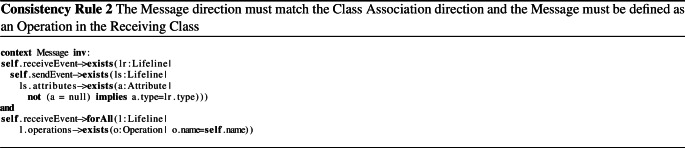


Consistency rules, however, can also be more complex as shown in CR 2, which checks that for every message in the sequence diagram, a corresponding association exists in the class diagram. Additionally, the rule also checks if the class diagram defines an operation with a corresponding name of the sequence diagram message. CR 2 is violated by messages *stop* and *wait* being called from an instance of *Streamer* to an instance of *User* (Fig. 1b). This message call is inconsistent because only the class *User* may call operations from *Streamer*, as shown in Fig. 1a. In addition, the messages *pause* and *wait* are inconsistent because they do not have a corresponding operation in their class (Fig. 1a), *i.e.*, an operation with name *pause*, another with name *wait*. Since CR 2 is a conjunction, violating at least one argument of the conjunction makes the rule evaluation inconsistent. However, we can already see that the reasons for the inconsistencies differ depending on which messages the rule is applied to. For instance, message *stop* violates the first conjunction only, message *pause* violates the second conjunction only, and message *wait* violates both conjunctions. Thus, it is intuitive that understanding the cause of an inconsistency is important to guide the developer toward its repair.

### Definitions

#### Definition 1

(*Model*) A model ${\mathbb {M}}$ consists of model elements ($me \in {\mathbb {M}}$) which contain properties (*py*). A property of a model element is referred to by element dot (.) property name, *e.g.*, “Streamer.name”. A property can be of ($\rightarrow$) a primitive type (*e.g.*, Boolean, Integer, Float, or String) or a reference to other model elements. Therefore, model elements are instances (*inst*) of a specific type (*ty*) defined by the modeling language (ML). For example, the operation *play* in the class *Streamer* has properties such as name=“play” which is of the type String (see Fig. 1a).

$$\begin{aligned} {\mathbb {M}}&:= \bigcup me\\ ty&\in ML\\ me&inst ty\\ me.py&\rightarrow {\mathbb {M}}\cup \text {``any value''} \ inst \ ty \end{aligned}$$

#### Definition 2

(*Consistency Rule*) A consistency rule (${\mathbb {C}}{\mathbb {R}}$) is a condition defined for a context that must be fulfilled by the model. This condition (*cond*) evaluates to a Boolean value (${\mathcal {B}}$) as *true* (consistent) or *false* (inconsistent). A consistency rule is defined for a context (*ct*). The context is a meta-model element, which is a type of a model element.

$$\begin{aligned} {\mathbb {C}}{\mathbb {R}}:= & {} \langle ct,\,cond\rangle \\ cond(me)\mapsto & {} true \vee false|me \ inst \ ct \end{aligned}$$

The condition itself is a hierarchically ordered (tree-based) set of expressions ($\epsilon$), where the root expression corresponds to the condition as a whole and its sub-expressions correspond to parts of the condition. An expression identifies an operation (*op*), it has a single parent (except for the root expression $\epsilon _{0}$) and one or more arguments (*args*), an expected result (*exp*) and an evaluated result (*re*).

$$\begin{aligned} cond&:= \bigcup _{i=0}^{n}\epsilon _{i}|\left\{ \begin{array}{ll} \exists i,j:\epsilon _{j}\in \epsilon _{i}.args &{} \text {if }j>0,\, i\ne j\\ \not \exists i,j:\epsilon _{j}\in \epsilon _{i}.args &{} \text {if j}=0,\,\ \hbox {i}\ne \hbox {j} \end{array}\right. \\ \epsilon&:= \langle op,\,args,\,exp,\,re\rangle \end{aligned}$$

Recall that CR 1 is composed of two parts: an exists expression(-$>exists(...)$) and an *AND* expression composed of two sub-expressions: a not-equals ($<>$) and an equals expression ($=$). Both expressions have two children: The $<>$ sub-expression has *o*1 and *o*2 as children, while $=$ has *o*1.*name* and *o*2.*name*. These children are leaf expressions, which either access model elements or are constants.

#### Definition 3

(*Evaluation Tree*) An evaluation tree represents a consistency rule evaluated for a specific model element. For instance, there are three operations in Fig. 1a, hence there are three evaluations^3^. Each evaluation checks if a consistency rule’s condition evaluates to *true*. This can be done recursively for every expression/sub-expression of a condition. The root expression of a condition is always expected to evaluate to *true*; however, this expectation may change with sub-expressions, *e.g.*, *not expressions*, as shown in [44]. An evaluation tree mirrors the tree structure of the consistency rule’s condition. However, in case of iterations (*e.g.*, *exists* quantifier in CR 2) their (sub) tree structures repeat for every iteration. Hence, the evaluation tree is an exact log of each operation computed during the evaluation of a condition. As an example, Fig. 3 shows an evaluation tree for CR 2. This evaluation tree will be explained in detail in Section 4.

#### Definition 4

(*Repair Action*) A repair action (*ra*) defines a change of a model element property that resolves an inconsistency in part or full, *e.g.*, often multiple repair actions are needed to resolve an inconsistency. A repair action identifies the operation (*op*), the model element (*me*), the model element property (*py*), and, optionally, a concrete value (*v* which can be a model element $v \in {\mathbb {M}}$) to change the model element property. The following operations are possible: **add** a model element to the model or to a collection of model elements, **delete** (del) a model element from the model or from a collection of model elements, and **modify** (mod) a model element property to a given value. In addition there are the constraining changes: $=$, $\ne$, <, >, where, respectively, a property has to be equal to a value, different from a value, less than a value, or greater than a value. ${\mathbb {R}}{\mathbb {A}}$ is the set of all possible repair actions. An abstract repair action ($\overline{ra}$) is an action where no concrete value can be calculated ($v=?$).

$$\begin{aligned}&ra \in {\mathbb {R}}{\mathbb {A}} := \langle op,me.py,v \rangle |v\ne ?\\&\overline{ra} \in {\mathbb {R}}{\mathbb {A}} := \langle op,me.py,v \rangle |v=? \\&op \in \lbrace add,del,mod, =, \ne ,<,>\rbrace \end{aligned}$$

To illustrate, we can consider class *Streamer* in Fig. 1a and CR 1, which is evaluated to *false* on both operations *play*, because their names are not unique. A possible *ra* in this case would be to delete (*del*) one of the *play* operations ($me.py = Streamer.operations$) from the collection of operations. The value would be one of the operations *play* ($v = play$). Thus, the *ra* would be: (*del*, *Streamer*.*operations*, *play*).

#### Definition 5

(*Repair*) A repair is a non-empty set of repair actions (*ra*) that fixes a specific inconsistency *i* from the set of all possible inconsistencies ${\mathbb {I}}$. This set of repair actions (*ras*) may also contain abstract repair actions ($\overline{ra}$).

$$\begin{aligned} \langle i \in {\mathbb {I}}, ras \subseteq {\mathbb {R}}{\mathbb {A}} \rangle \end{aligned}$$

Considering CR 2 evaluation for message *wait*, the result is *false* (inconsistent) as it evaluates to *false* for both parts of the conjunction. To fix this inconsistency, a repair should have *ras* for fixing both parts of the conjunction. For the first part, message *wait* direction should be inverted, meaning that class *User* should be calling it from class *Streamer*. Thus, the *ra* should modify (*mod*) the message *receiveEvent* property. In this case, the *ra* would be: (*mod*, *wait*.*receiveEvent*). Then, a *ra* should *add* message *wait* to the *Streamer* class operations list. Finally, the *ra* would be: (*add*,  *Streamer*. *operations*,  *wait*). The repair for fixing this inconsistency would be composed of these two *ras*.

#### Definition 6

(*Repair Tree*) A repair tree is a hierarchical ordered set of repair nodes for a single inconsistency. The nodes of a repair tree define whether the underlying repairs are alternatives ($*$) or sequences ($+$). Repair alternative nodes follow the exclusive or-alternative (XOR) principle where, from a set of repair alternatives, only one must be selected at a time for fixing an inconsistency. Based on that, for selecting a different alternative, the user should undo the previous one. Sequence repair nodes, however, indicate that all those repairs actions should be performed to fix the inconsistency. For instance, if we consider CR 1, for fixing the inconsistency on messages *play*, we could either delete (*del*) one of the messages or modify its name ($v \ne$ *“play”*). Thus, our repair tree would have an alternative repair node (*) with two possible repairs. More details are discussed in Sect. 4.

### Ownership-based filter

For reducing the possible combination of repair actions from the repair tree, we propose filtering repairs based on the ownership of model elements. In this paper, we define two types of ownership: artifact-based ownership and user-based ownership. Considering the artifact-based ownership, depending on the consistency rule, a repair action may suggest modifying model elements from different artifacts, *e.g.*, an operation from a class diagram, or a message from a sequence diagram. We consider these model elements to be owned by this artifact, as they belong to it. In UML, for example, this ownership may be based on diagrams, *e.g.*, operation *stop* is owned by the class diagram (Fig. 1a), while message *pause* is owned by the sequence diagram (Fig. 1b). This allows our approach to filter repair actions based on this ownership.

To better understand this scenario, firstly, let us consider CR 2. As discussed earlier, message *wait* violates this CR in both parts of the conjunction. To fix the first part of the conjunction, *i.e.*, association direction must match message direction, a repair action (*ra*) could suggest changing the direction of the message on the sequence diagram (*ra*1) or changing the association direction on the class diagram (*ra*2). For the second part of the conjunction, *i.e.*, message must be defined as an operation, a *ra* could suggest renaming message *wait* on the sequence diagram to “*play*” (*ra*3) or adding an operation called “*wait*” on the class diagram (*ra*4).
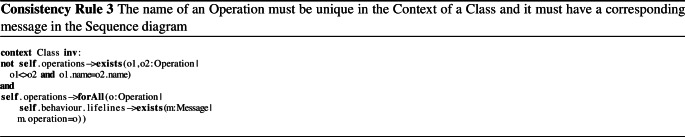


Note that for fixing this inconsistency, two repair actions must be performed, and the possibilities are *ra*1 and *ra*3, *ra*1 and *ra*4, *ra*2 and *ra*3, or *ra*2 and *ra*4. However, if we consider the type of the diagram as a filtering mechanism for reducing the possible repairs, we can focus on repairing the inconsistency only in the sequence diagram. Thus, repair actions suggesting changes in the class diagram would be filtered out of the possibilities. In this case, a possible repair would be to execute *ra*1 and *ra*3, reducing the list of possible repairs.

The aforementioned scenario presents an opportunity for simplifying the repair tree by filtering out repair actions originating from different diagrams. Removing these repair actions, however, could also mean that the inconsistency might not be fixable. To understand this, let us consider CR 3 that also contains a conjunction. The first part evaluates if an operation on a class has a unique name, while the second part evaluates if there is a message on the sequence diagram for all operations in a class. In this case, operation *play* would be evaluated to *false* on both conjunctions as its name is not unique (Fig. 1a) and it has no corresponding message (Fig. 1b). Possible repair actions for fixing the first part of the conjunction would be to change the name of the first operation *play* (*ra*1) or deleting the second operation *play* (*ra*2). For the second part of the conjunction, a message corresponding to the operation play could be added in the sequence diagram (*ra*3), or the message *pause* could be renamed to “play” (*ra*4). Similarly to CR 2, for fixing this inconsistency, both parts of the conjunctions must be fixed. Thus, possible repairs would be *ra*1 and *ra*3, *ra*1 and *ra*4, *ra*2 and *ra*3, or *ra*2 and *ra*4. If we consider filtering repair actions that only affect the sequence diagram, however, none of these repairs would be possible. This happens because for fixing this inconsistency, model elements owned by both diagrams must be changed.

In the aforementioned example, the total number of repairs is not big. However, depending on the rule and the model the number of model elements being accessed by the evaluation tree can grow exponentially [44], for instance, when using the iterative expression *forAll* to evaluate a large set of properties. Thus, the number of repairs for fixing a single inconsistency can be large. This would overwhelm the developers as they would need to decide on executing a repair among hundreds or even thousands of options. Therefore, while still beneficial as it could reduce the amount of possible repairs, the filtering has to be handled carefully to prevent not fixing the inconsistency.

Our approach also considers user-based ownership for filtering repairs. In this context, the user-based ownership describes which developers are the *owners*, thus having reading and writing privileges of a model element. In this paper, we consider that a model element may have several *owners*. The reason why a developer owns a model element is not significant in this work, since companies using our approach can have different ownership strategies. For example, the ownership may be based on the type of UML diagrams, which would be similar to the scenario described earlier. This ownership could also be defined in terms of features, which would make users own several model elements across different diagrams.

By considering the user-based ownership aspect, we argue that developers may only be interested in repair actions that affect their owned model elements. In this sense, we can consider filtering repair actions based on the user ownership of the model elements being modified. However, this could lead to a problem similar to the artifact-based ownership, as the repair tree may end up not fixing the inconsistency. Considering the repair of message *wait* on CR 2, if a developer has no ownership rights in the class *Streamer*, repair actions for fixing the second part of the conjunction could not be performed. However, the opposite could also happen as a large set of repairs could be generated. This would make the developer choose among hundreds of repairs, which can be overwhelming. Thus, we argue that filtering repairs can reduce this number, aiding the developer on the decision-making.

The user-based ownership may be limited due to companies not applying this type of restriction in their artifacts, especially UML models. However, when we consider source code, developers are responsible for their part of the source code. For example, we have worked with companies from the electrical engineering domain which collaborate with software engineers for modeling and implementing robotic components. In this case, ownership was important because engineers from different domains usually only work with the models from the domain that they are familiar with. Furthermore, there may be no repairs modifying elements exclusively owned by a single engineer. Hence, the user-based ownership can be beneficial by allowing the engineer to know who are the other engineers that must collaborate to fix the inconsistency..

We address the ownership filtering for both types of ownership, *i.e.*, artifact-based and user-based (see Sect. 4.10). This is performed by highlighting repair actions in the repair tree according to the ownership of model elements being modified. In addition, we discuss how we provide a set of possible repairs generated from the repair tree, using a highlighting mechanism for filtering them.

## Goal and contribution

By focusing on the causes of inconsistencies, our approach identifies the model element properties that must be repaired. The cause of an inconsistency consists of model element properties that contribute to that inconsistency ($cond(me)=false$) [44]. We argue that an inconsistency can be repaired only by changing at least one of the model element properties of the cause. Hence, repairs based on the cause must still enumerate all repair alternatives. The focus on the cause gives developers a complete picture of the breadth of the inconsistency problem and all possible repair alternatives that should be considered at this point. It is important to mention that the cause of inconsistencies is almost always a subset of the model elements involved in the computation of inconsistencies [44]. However, this depends on the expressivity of the consistency rules being used for evaluating the consistency of the model. Thus, there is the possibility of rules that are not expressive enough, *e.g.*, a rule that checks all model elements in a model, and can induce our approach to provide a subset of repairs with all possible repair alternatives.

The focus on fast and incremental repairs is an important part of the goal to ensure that developers continuously have access to up-to-date repairs. This has several benefits: (i) The developer is not forced to repair an inconsistency immediately, but the repairs are kept updated, and (ii) the approach helps the developer explore the side effects of repairs incrementally because once a repair is chosen by the developer, its side effects are immediately computed and reflected in the new set of repairs. In that regard, our approach helps to guide the developer to explore the depths of an inconsistency problem.

Based on the goal of this work, this paper proposes an approach that:

1. generates abstract repairs for causes of inconsistencies,
2. structures repairs into repair alternatives and sequences using repair trees,
3. supports arbitrary, user-definable consistency rules,
4. is fast, incremental, and scalable as repairs are computed instantly when the model changes,
5. supports highlighting repair actions based on the model elements ownership, using this for filtering out repairs, and
6. supports consistency checking in different types of models, providing evidence of its use with UML and Java source code.

While contributions 1-4 are reported in previous work [42–44]. In this paper, we extend the discussion on repair generation by describing how to generate repairs for additional operations in comparison to previous work [44], including non-Boolean operations (see Sect. 4.8). Furthermore, contributions 5 and 6 extend previous work, as in this paper we apply a highlighting and filtering mechanism for repairs based on ownership of the model elements being modified by the repair actions (see Sect. 4.10). Also, we implemented our approach into a supporting tool called DesignSpace-IR. This tool provides support for consistency checking and repair generation to UML design models as already present in the Model/Analyzer tool [42], as well as for different types of models (see Sect. 4.11).

In addition to these contributions, this paper also reports an empirical evaluation for measuring the scalability and usability of our approach as well as the highlighting and filtering mechanisms.

## Approach

In this section, we present our approach. Firstly, we introduce the principle of computing the cause of the inconsistency. Then, we discuss how we generate the evaluation tree, expected results, and repair tree. Next, we present the repair generator functions, including those for non-Boolean expressions. We also discuss how we simplify a raw repair tree by removing redundancies. Finally, we introduce the principle of filtering repairs based on ownership of model elements, as well as describing how our tool provides support for different models.

### Principle

**Fig. 2:**
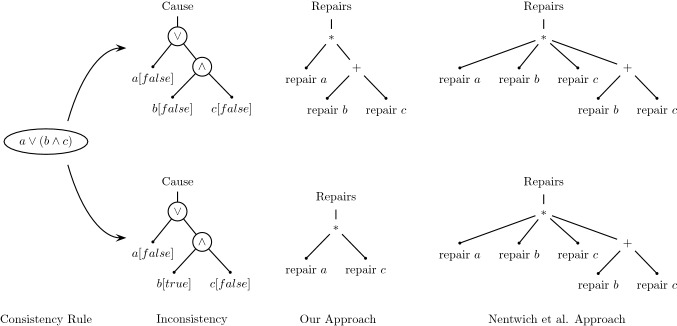
Our Approach computes different Repair Trees for Consistency Rule $a\vee (b\wedge c)$ depending on the actual evaluation results of *a*, *b*, or *c* (the causes). Compare bottom and top. The figure depicts the causes, the repair trees generated by our approach, and the repair trees generated by Nentwich et al. [33] which is the same in both cases as their approach does not consider the cause. Our approach’s focus on the causes results in smaller repair trees that avoid unnecessary repair actions not relevant to the inconsistency at hand

Our approach analyzes the evaluation of an inconsistency to generate repairs. Consider the consistency rule $a\vee (b\wedge c)$ where *a*, *b*, and *c* are Boolean expressions on a model. If inconsistent, any combination of these Boolean expressions may need repairing. Theoretically possible repair alternatives are the power set: $\{a\}$, $\{b\}$, $\{c\}$, $\{a,b\}$, $\{a,c\}$, $\{b,c\}$, $\{a,b,c\}$. For example, repair $\{b\}$ suggests a way of changing the model elements referred to by *b* such that validating *b* becomes *true*. Recall that CR 2 was essentially a conjunction (like $b\wedge c$). Hence, repair $\{b\}$ could imply repairing an argument of that conjunction, *e.g.*, self.receiveEvent->exists($\ldots {}$), which may require repairing one or more model element properties accessed by that argument. A repair alternative may also contain sequences of repair actions. For example, repair $\{b,c\}$ is a sequence of repair $\{b\}$ and repair $\{c\}$. It is also possible that repair alternatives overlap in their repair actions, *e.g.*, repair $\{a,b,c\}$ overlaps with repair $\{b,c\}$.

Nentwich et al. [33] recognized that developers do not need to investigate the entire power set of repair alternatives. For example, repair $\{a\}$ would make $a\vee (b\wedge c)$ consistent regardless of repairs $\{b\}$ or $\{c\}$. Hence, by considering the structure of the consistency rule condition, they eliminate unnecessary sequences of repair actions, such as repair $\{a,b\}$, $\{a,c\}$, and $\{a,b,c\}$. The list of still complete repair alternatives for this consistency rule condition reduces to: $\{a\}$, $\{b\}$, $\{c\}$, or $\{b,c\}$. However, this list applies to the consistency rule as a whole and is not yet customized to a particular inconsistency at hand. There are still incorrect repair alternatives. For example, let us assume that *b* is *true* already, while *a* and *c* are *false*. In this case, $a\vee (b\wedge c)$ would be inconsistent but only two repair alternatives out of the four mentioned would be feasible: repair $\{a\}$ or repair $\{c\}$. Repair $\{b\}$ is not necessary because *b* is already *true*. And repair $\{b,c\}$ would be non-minimal because it would force the developer to also repair $\{b\}$. This is where our work extends Nentwich et al. [33] because we also consider the cause of the inconsistency, *i.e.*, why the inconsistency happened.

It is important to understand that the repair alternatives of a given consistency rule may differ depending on the situation at hand. For example, if all three expressions *a*, *b*, and *c* were *false*, then the correct list of repair alternatives would be: repair $\{a\}$ or repair $\{b,c\}$. In fact, there is no combination of *a*, *b* and *c* being *true* or *false* that would require all four repair alternatives $\{a\}$, $\{b\}$, $\{c\}$, and $\{b,c\}$ as suggested by Nentwich et al. [33], though their approach generates an upper bound of all possible repairs. Their approach is thus complete; however, it does contain irrelevant repair alternatives when applied to specific situations. By investigating the cause of an inconsistent design, we are able to reduce the list of repair alternatives. However, the list of repair alternatives may still be verbose because repair actions often overlap among repair alternatives. These two situations discussed are depicted in Fig. 2. The constraint’s syntactic structures with evaluation results are depicted on the left and the corresponding repair trees computed by our approach are depicted in the middle of the figure. For $a=b=c=false$ (top), the possible repair alternatives are: repair $\{a\}$ *or* repair $\{b\ and \ c\}$. For $a=c=false$ and $b=true$ (bottom) the possible repair alternatives are: repair $\{a\}$ *or* repair $\{c\}$. The figure also depicts the equivalent repair tree by Nentwich et al. [33] (right) which only considers the rule structure but not the cause. Hence, their approach suggests the same repairs regardless of the cause of the inconsistency. Their approach is also a worst-case super-set of repairs that consider the cause.

### Generation of the evaluation tree

The evaluation tree represents the detailed results of the evaluation of a consistency rule. The evaluation tree is essentially a hierarchical log of a consistency rule’s evaluation, complete with all expressions that were evaluated and their observed, intermediate evaluation results. The evaluation tree forms the foundation for computing the repair tree. For compactness and to show the general applicability of our approach to any constraint language that follows first-order logic (cf. xLinkit [32] and Beanbag [54]), we use the mathematical notation for the consistency rule condition instead of the OCL notation, *i.e.*, $\exists \longleftrightarrow$ exists. For example, the CR 2 expressed in first-order logic is presented as:

$$\begin{aligned}&{ self}\mapsto { Message}:\\&(\exists lr\in { self}.{ receiveEvent}.{ covered}|\\&\quad \exists ls\in { self}.{ sendEvent}.{ covered}|\\&\qquad \exists a\in ls.{ represents}.{ type}.{ ownedAttribute}|\\&\quad \qquad \lnot (a=null)\Rightarrow a.{ type}=lr.{ represents}.{ type})\\&\wedge \\&(\forall l\in { self}.{ receiveEvent}.{ covered}|\\&\quad \exists o\in l.{ represents}.{ type}.{ ownedOperation}|\\&\qquad o.{ name}={ self}.{ name}) \end{aligned}$$

The evaluation tree contains a node for every evaluated expression and the nodes are annotated with their expected and evaluated results. Algorithm 1 summarizes how an evaluation tree is build up during the evaluation of a consistency rule condition. The algorithm starts with the consistency rule and the context elements as input (Line 1). The context element is the variable “self” used in the consistency rules, the “evaluate” algorithm executes the consistency checker on the root element of the rule’s condition (Line 2), and the “expected” algorithm sets the expected results (Line 3). For example, for CR 2 evaluated on message *wait* the root expression is a conjunction and this conjunction is evaluated first (Lines 6-29). The algorithm starts by creating a node for each expression evaluated (Line 7). If the expression is the root expression, then its node is remembered (Line 7); otherwise, the created node is added as a child to the parent node (Line 9). The algorithm then evaluates the operation of the expression. As there could be many kinds of operations, we only present some operations commonly used in this paper, depicted in Lines 10-25. For example, a conjunction is evaluated by recursively calling the “evaluate” algorithm on both its arguments (Lines 12-13) followed by applying the logical *and* operator and their argument results. There are two important observations here: (i) The recursive evaluation always takes the current node as the next parent node, and (ii) the result of the evaluation is stored in the node’s evaluated field^4^. The evaluation of other operations is quite analogous and a few more examples are provided in the algorithm. However, do note that the evaluation tree is not a syntax tree. This is most evident for quantifiers. For example, the *forall* quantifier (Lines 20–26) has two arguments: the first to identify a collection of elements and the second to describe the condition that must hold for every element in the collection. This quantifier is thus evaluated on as many elements as are provided in the collection. Hence, the evaluation tree creates as many nodes as there are elements to evaluate (zero to many) and each node’s sub-tree describes the evaluation of the condition on that element.
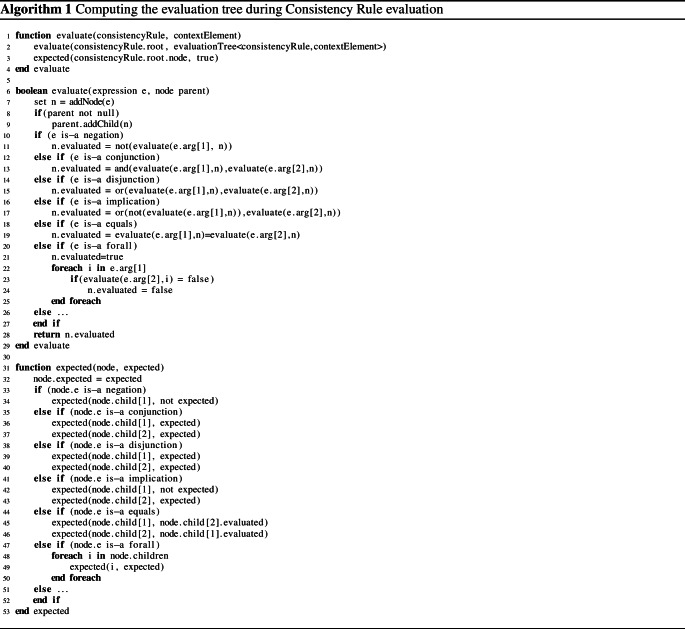


### Generation of expected result

It is important to distinguish between the expected and evaluated result because an inconsistency is caused only if the two differ [44]. The “evaluate” algorithm builds up the evaluation tree with all its evaluated results, *i.e.*, n.evaluated. Expected results are computed by the “expected” algorithm (Lines 31–53). The “expected” algorithm recursively traverses the evaluation tree from top to bottom and passes the expected results of the parent node to its children (Line 32). The expected result is always *true* for the root node (Line 3). For all its sub-nodes, the expected result must be computed depending on the node’s operation. For example, the arguments of a conjunction are expected to evaluate to the same result as the conjunction itself. For $(a\wedge b)$ to be *true*, both *a* and *b* are expected to evaluate to *true* also (Lines 35–37). However, not every conjunction must evaluate to *true*. For example, consider $\lnot (a\wedge b)$. Here, the negation of the conjunction implies that the conjunction is expected to evaluate to *false*. The negation node is a parent of the conjunction node. The negation is expected to evaluate to *true*, and the “expected” algorithm changes this to *false* for its child node (Lines 33-34). The subsequent call of “expected” on the conjunction thus expects the conjunction to be *false*. (The expected argument would be *false*.) At least one node of the conjunction is then expected to be *false* also.

**Fig. 3:**
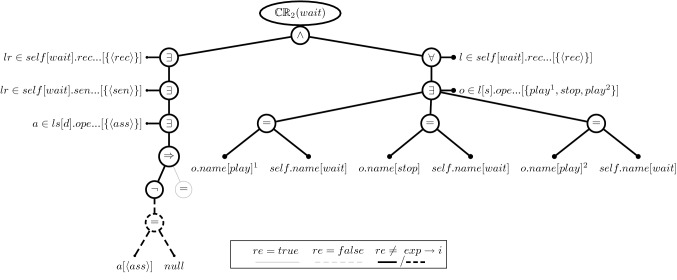
Evaluation Tree for the Inconsistency of Consistency Rule 2 on Message *wait*

Furthermore, the expected result is not the same as the cause of a repair. The expected result is determined for each argument separately as implying: What Boolean state is expected of the argument for it to not cause an inconsistency. Consider, for example, the disjunction $a\vee b$. Only one of the two argument needs to be *true* for the disjunction to be *true*. Still, both arguments are expected to be *true* because for computing repairs we have to understand the role of each argument individually. So, if *a* in $a\vee b$ should be repaired, then it ought to be repaired to *true*. The same is *true* for *b* in $a\vee b$. This rationale also explains the implication $a\Rightarrow b$ where *a* is expected to be *false* and *b* is expected to be *true*, meaning: If *a* in $a\Rightarrow b$ should be repaired, then it ought to be repaired to *false* because the implication would be *true* regardless of *b*. Or, if *b* in $a\Rightarrow b$ should be repaired, then it ought to be repaired to *true* because if *b* matters, then it must be *true*. Most expected results are passed from parents to children but there are exceptions. For example, for the equality $a=b$ to be *true*, the equality’s argument *a* is expected to match its sibling argument *b* and vice versa (Lines 44-46).

### Evaluation tree structure

Let us return to the illustration and the aforementioned CR 2. Figure 3 shows its evaluation tree if evaluated on message *wait*. The notation we use in this figure and all the following figures is that the expected result is indicated as solid edges for *true* and dashed edges for *false*. If the evaluated and expected results are equal, the edges are drawn in gray thin lines; if they differ, then the expected results are drawn in black thick lines. The black thick lines thus depict the cause of an inconsistency. The values of the variables and properties are given in square brackets and property values without names are in angle brackets, *e.g.*, $\langle rec\rangle$ represents the receive event element of type MessageOccurenceSpecification.

The root represents the consistency rule evaluation and has exactly one node which represents the conjunction of the rule as was discussed earlier. The left-hand side (left argument) is the rule’s existential quantifier that iterates over the lifelines the message is sent to. The source argument of a quantifier is a horizontal branch ($lr\in self[wait].rec\ldots$). In our example, there is only one lifeline and therefore only one branch for the quantifier condition is created. Concerning the quantifiers, the first one iterates over the receivers of a message and the second one iterates over its senders. The third and final existential quantifier iterates over the associations between the sender and receiver ($a\in ls[d].ope\ldots$). Its condition is an implication that has as its left-hand argument an inequality relation. This relation evaluates if the attribute of the sender lifeline is not null and, if it is not null, the right-hand argument of the implications evaluates if the attribute type of the sender lifeline is equal to the type of the receiver lifeline. In the *wait* message, the attribute is null and therefore the right-hand argument is an empty equality relation with no arguments. With the left-hand argument of the implication, we see a case where the expected result for an expression is not *true* because the negation expects that the attribute *a* must not be null (see dashed-black lines in lower-left of Fig. 3).

**Fig. 4:**
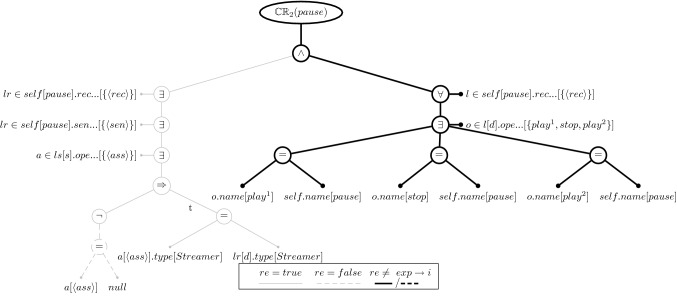
Evaluation Tree for the Inconsistency of Consistency Rule 2 on Message *pause*

The right-hand argument of the root expression (the conjunction) starts with an universal quantifier that iterates over the receiver lifelines ($l \in self[wait].rec\ldots$). As we already know, there is only one element that satisfies that quantifier condition. The existential quantifier below thus iterates over this one element which references the operations of that element’s owner: the class of the receiver lifeline object. This class, *Streamer*, has three operations, and therefore, the condition has to be evaluated three times. The leaves of the evaluation tree are expressions that access model element properties. For example, the source expression of the existential quantifier on the left-hand argument and the universal quantifier on the right-hand argument of the root expression access the same model element properties: first the property *receiveEvent* of the message *wait* and then the *operations* property of the element returned by the first property call. The result is a collection of property calls of the message and operation names that are the leaves of the right-hand argument of the conjunction. Since none of the three operations is named *wait*, all three equality relation evaluate to *false*. However, as the quantifier is expected to evaluate to *true*, it is also expected that at least one of its conditions evaluates to *true*. Since this is not the case, all three equality relations are part of the cause of the inconsistency (*i.e.*, any one of them could have satisfied the expected result). In this example, nearly the entire evaluation tree causes the inconsistency because message *wait* violates both conjunctions. Hence, much of the evaluation tree is painted in black, thick lines.

The evaluation tree reflects one-to-one the execution of the consistency rule of the message *wait*, similar to a hierarchical log of a program execution. Naturally, the evaluation of the same rule on another message may result in a different evaluation tree because: (i) the quantifiers may have different collections (*e.g.*, more or fewer operations), (ii) the concrete model elements accessed may be different, or (iii) the evaluation results of the various expressions may be different. Hence, the evaluation tree of CR 2 evaluated on message *pause* (Fig. 4) looks somewhat different from the evaluation tree for message *wait* (Fig. 3), even though the consistency rule is the same. Figure 4 shows that the message *pause* violates the second conjunction only, resulting in the right-hand side of the evaluation tree to be painted in black, thick lines only. The receiving object’s class is *Streamer*, and its operations would lead to sub-trees on the right-hand side of the evaluation tree, *i.e.*, all of which causing the inconsistency. Moreover, many of the leaves point to different model elements. The evaluation tree thus reflects the concrete situation of an inconsistency which is important for generating appropriate repairs. This is discussed next.

### Repair tree structure

The cause of an inconsistency is determined through the evaluation tree and it is the basis for the repair tree. Thus, if a node in the evaluation tree is part of the cause, then it can be repaired. The repair of a node usually requires the repair of all its arguments that are also part of the cause. There are exceptions, however, where we are not required to repair all arguments, *e.g.*, disjunctions, or existential quantifiers. Besides that, the repair tree would still have repair nodes for all the arguments as alternatives for the developer. These alternatives, however, would have an exclusive relation where only one should be selected.

Based on a conjunction, we show how repair alternatives are generated if an expression $a\wedge b$ causes an inconsistency. Table 1 shows the four possible cases of an inconsistency caused by this expression. If $a\wedge b$ is expected (*exp*) to be *true* for the consistency rule, then a repair is only needed if $a\wedge b$ evaluates to *false* ($re=a\wedge b$). There are three cases when this may happen: when either *a* is *false*, or *b* is *false*, or both *a* and *b* are *false*. Hence, the repair alternatives are repair $\left\{ a\right\}$ or repair $\left\{ b\right\}$ or repair $\{ a + b\}$ (a sequence of repairs where the order is irrelevant). However, if $a\wedge b$ is expected to be *false*, *e.g.*, $\lnot (a\wedge b)$, then a repair is only needed if $a\wedge b$ evaluates to *true* and there is only one case when this may happen: when both *a* and *b* are *true*. Here, the only repair alternative is repair $\{ a * b\}$ (an exclusive-or where only one must be selected). This example also illustrates the need for expected and evaluated results because negations in conditions change the expected outcome.

Repairs derived from the cause do not consider side effects, *i.e.*, the repairing of the cause may inadvertently break something else or reveal something else that is broken. For example, repairing *a* may break *b*. Indeed, there often are infinite such side effects if we consider the repair an open world problem, a problem were developers may add new information not yet present in the model, *e.g.*, adding a new operation for class *Streamer* that will have the message’s name; or a new parent class to *Streamer* which has a new operation, and so on. The focus on the immediate cause deals with what must be done rather than what may or may not happen afterward. It must be noted that understanding side effects is important but so is understanding the cause. We argue that it is not possible to repair an inconsistency if it does not repair the cause. Repairing the cause thus explores the breadth of repairs for a given inconsistency. If we then proceed with the repair and the repair indeed breaks something else or reveals another problem, then our approach will update the evaluation tree/repair tree to reflect the new situation. For example, if repairing *a* breaks *b*, then after repairing *a* the evaluation tree will update based on new cause (*b*). Thus, our approach will suggest to repair *b* at that point, an incremental characteristic. It is the role of the developer to guide the repair. Note that deleting *a* and *b* are not valid repairs here because we want to repair model elements, not consistency rules.

**Table 1:** Repair Alternatives for $a\wedge b$ depending on Expected and Evaluated Results

$\#$	*a*	*b*	*exp*	$re=a\wedge b$	*R*
1	*false*	*true*	*true*	*false*	$\left\{ a\right\}$
2	*true*	*false*	*true*	*false*	$\left\{ b\right\}$
3	*false*	*false*	*true*	*false*	$\left\{ a+ b\right\}$
4	*true*	*true*	*false*	*true*	$\left\{ a* b\right\}$

### Repair tree generation

Algorithm 2 shows how the repair tree is generated. As input, it takes the evaluation tree of an inconsistent consistency rule. The generation starts with the root expression of the evaluation tree (Lines 2-3). First the function “TableLookUp” is called to determine how the expression has to be repaired (Line 7). The function “TableLookUp” is a lookup onto Table 2, which contains repairs for individual operations. This function returns two values: (i) a repair node (an alternative $*$ or sequence $+$) and (ii) the subset of arguments that are part of the cause and also require repairing (*causeArgs*). This subset can contain further expressions that need to be handled recursively (Line 11) or it may contain repair actions (Line 13).
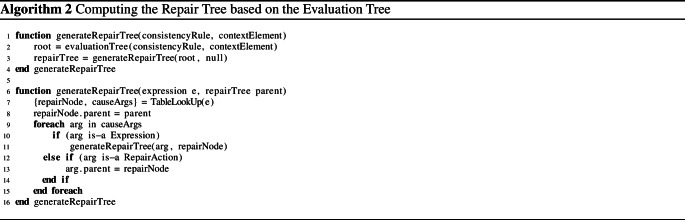


Table 2 depicts the various operations of the consistency rule language and how they can be repaired if violated. The second column lists the expression type (operation), and the third column presents the repair generator functions with their guard conditions. To understand the notation used, consider the conjunction #2 in Table 2, which lists all four repair alternatives we previously discussed in Table 1. For example, we see that the function “TableLookUp” returns $\{+,\{b\}\}$ if the expected result is *true* ($exp=t$), the first argument *a* was evaluated to *true* ($re_{a}=t$), and the second argument *b* was evaluated to *false* ($re_{b}=f$). This is equivalent to alternatives #2 in Table 1 with the only difference that we explicitly define the type of the repair node to be a sequence ($+$), which is a sequence of one repair only.

Once Algorithm 2 retrieves the repair node and the arguments that are part of the cause of the inconsistency, it attaches the retrieved repair node to its parent node which was given as a parameter to the algorithm (Line 8). Then, for each argument that is part of the cause of the inconsistency, the “generateRepairTree” function is called recursively to add to the repair tree. The arguments to the recursive call are i) the expression of the argument, and ii) the current repair node that will become the parent of any recursively added repair node (Lines 10-11). The algorithm thus navigates the subset of the evaluation tree that causes the inconsistency and it attempts to repair each node of the cause through a corresponding node in the repair tree. The repairs in Table 1 are thus guarded by “if” statements to describe what repair is necessary depending on the cause.

**Table 2:** Excerpt of Rules for the Generation of the Repair Tree

	$\epsilon$	$\{RepairNode,causeArgs\}$
#1	$\lnot a$	$\langle +,\{(a,\lnot exp)\}\rangle$
#2	$a\wedge b$	$${\left\{ \begin{array}{ll} \langle +,\{a\}\rangle &{} \text {if }exp=t,\,re_{a}=f,\,re_{b}=t\\ \langle +,\{b\}\rangle &{} \text {if }exp=t,\,re_{a}=t,\,re_{b}=f\\ \langle +,\{a,b\}\rangle &{} \text {if }exp=t,\,re_{a}=f,\,re_{b}=f\\ \langle *,\{a,b\}\rangle &{} \text {if }exp=f,\,re_{a}=t,\,re_{b}=t \end{array}\right. }$$
#3	$a\vee b$	$${\left\{ \begin{array}{ll} \langle *,\{a,b\}\rangle &{} \text {if }exp=t,\,re_{a}=f,\,re_{b}=f\\ \langle +,\{a\}\rangle &{} \text {if }exp=f,\,re_{a}=t,\,re_{b}=f\\ \langle +,\{b\}\rangle &{} \text {if }exp=f,\,re_{a}=f,\,re_{b}=t\\ \langle +,\{a,b\}\rangle &{} \text {if }exp=f,\,re_{a}=t,\,re_{b}=t \end{array}\right. }$$
#4	$a\oplus b$	$${\left\{ \begin{array}{ll} \langle *,\{ a ,b \}\rangle &{} \text {if }exp=t,\,re_{a}=f,\,re_{b}=f\\ \langle *,\{(a ,\lnot exp),(b ,\lnot exp)\}\rangle &{} \text {if }exp=t,\,re_{a}=t,\,re_{b}=t\\ \langle *,\{( a ,\lnot exp),b \}\rangle &{} \text {if }exp=f,\,re_{a}=f,\,re_{b}=t\\ \langle *,\{a ,(b ,\lnot exp)\}\rangle &{} \text {if }exp=f,\,re_{a}=t,\,re_{b}=f \end{array}\right. }$$
#5	$a\Rightarrow b$	$${\left\{ \begin{array}{ll} \langle *,\{a,b\}\rangle &{} \text {if }exp=t,\,re_{a}=t,\,re_{b}=f\\ \langle +,\{b\}\rangle &{} \text {if }exp=f,\,re_{a}=t,\,re_{b}=t\\ \langle +,\{a,b\}\rangle &{} \text {if }exp=f,\,re_{a}=f,\,re_{b}=t\\ \langle +,\{a\}\rangle &{} \text {if }exp=f,\,re_{a}=f,\,re_{b}=f \end{array}\right. }$$
#6	$a=b$	$${\left\{ \begin{array}{ll} \langle *,\{(mod,a,b),(mod, b, a)\}\rangle &{} \text {if }exp=t\\ \langle *,\{(mod,a,\ne b),(mod, b, \ne a)\}\rangle &{} \text {if }exp=f \end{array}\right. }$$
#7	$a \ge b$	$${\left\{ \begin{array}{ll} \langle *,\{(mod, a,> b),(mod, a, b),(mod, b ,< a),( mod, b , a)\}\rangle &{} \text {if }exp=t\\ \langle *,\{(mod, a , < b),(mod, b , > a)\}\rangle &{} \text {if }exp=f \end{array}\right. }$$
#8	$a \le b$	$${\left\{ \begin{array}{ll} \langle *,\{(mod, a,< b),(mod, a, b),(mod, b,> a),(mod, b, a)\}\rangle &{} \text {if }exp=t\\ \langle *,\{( mod, a, > b),(mod, b, < a)\}\rangle &{} \text {if }exp=f \end{array}\right. }$$
#9	$\forall a\in A:b$	$${\left\{ \begin{array}{ll} \langle +,\{\bigcup _{i=1}^{|A|}\{\langle *,\{(del, A ,b_{i}),b_{i}\}\rangle |re_{bi}=f\}\}\rangle &{} \text {if }exp=t\\ \langle *,\{(add, A, ?),\langle *,\{\bigcup _{i=1}^{|A|}\{b_{i}\}|re_{bi}=t\rangle \}\rangle &{} \text {if }exp=f \end{array}\right. }$$
#10	$\exists a\in A:b$	$${\left\{ \begin{array}{ll} \langle *,\{\langle add,\, A,\, ?\rangle ,\langle *,\{\bigcup _{i=1}^{|A|}\{b_{i}\}|re_{bi}=f\rangle \}\rangle &{} \text {if }exp=t\\ \langle +,\{\bigcup _{i=1}^{|A|}\{\langle *,\{(del, A, b_{i}),b_{i}\}\rangle |re_{bi}=t\}\}\rangle &{} \text {if }exp=f \end{array}\right. }$$
#11	*a*.*b*	$\langle *,\{( mod, a),\, b\}\rangle$
#12	*A*.*isEmpty*	$${\left\{ \begin{array}{ll} \langle +,\{\bigcup _{i=1}^{|A|}\{\langle +,(del,A, a_{i})\rangle \}\}\rangle &{} \text {if }exp=t\\ \langle +,\{\langle add,\, A,\, ?\rangle \}\rangle &{} \text {if }exp=f\\ \langle +,\{\bigcup _{i=1}^{|A|}\{\langle *, \{(del,A,a_{i}), (mod, a_{i}, \lnot re_{ai})\}\rangle \}\}\rangle &{} \text {if }exp=t \ and \ A \in \epsilon \\ \langle *,\{\bigcup _{i=1}^{|A|}\{b_{i}\}|re_{bi}=t\}\rangle &{} \text {if }exp=f \ and \ A \in \epsilon \end{array}\right. }$$
#13	*A*.*includes*(*b*)	$${\left\{ \begin{array}{ll} \langle +,\{(add, A, b)\}\rangle &{} \text {if }exp=t\\ \langle +,\{(del, A, b)\}\rangle &{} \text {if }exp=f \end{array}\right. }$$
#14	$A.includesAll(b \in B)$	$${\left\{ \begin{array}{ll} \langle +,\{\bigcup _{i=1}^{|B|}\{\langle *,\{(add, A, b_{i}),(del, B, b_{i})\}\rangle \}\}\rangle &{} \text {if }exp=t\\ \langle +,\{\bigcup _{i=1}^{|B|}\{\langle *,\{(del, A, b_{i}) , (add, B, ?)\}\rangle \}\}\rangle &{} \text {if }exp=f \end{array}\right. }$$
#15	*A*.*size*() : *a*	$${\left\{ \begin{array}{ll} \langle +,\{\bigcup _{i=re_{a}}^{|exp+1|}\{\langle +,(add, A, ?)\rangle \}\}\rangle &{} \text {if }op \ is =, exp> re_{a}\\ \langle +,\{\bigcup _{i=exp}^{|re_{a}+1|}\{\langle +,(del, A, ?)\rangle \}\}\rangle &{} \text {if }op \ is =, exp< re_{a}\\ \langle *,\{\langle (add,\, A,\, ?), (del,\, A,\, ?)\rangle \}\rangle &{} \text {if }op \ is \ne \\ \langle +,\{\bigcup _{i=re_{a}}^{|exp|}\{\langle +,(add, A, ?)\rangle \}\}\rangle &{} \text {if }op \ is >\\ \langle +,\{\bigcup _{i=exp}^{|re_{a}|}\{\langle +,(del, A, ?)\rangle \}\}\rangle &{} \text {if }op \ is <\\ \end{array}\right. }$$
#16	*a*.*subString*(*x*, *y*)	$${\left\{ \begin{array}{ll} \langle +,(mod, a, ?)\rangle &{} \text {if }op \ is \ = \\ \langle +,(mod, a, \ne a)\rangle &{} \text {if }op \ is \ \ne \end{array}\right. }$$
#17	*a*.*concat*(*x*)	$${\left\{ \begin{array}{ll} \langle *,\{(mod, a, ?), (mod, x, ?)\}\rangle &{} \text {if }op \ is \ = \\ \langle *,\{(mod, a, \ne a), (mod, x, \ne x)\}\rangle &{} \text {if }op \ is \ \ne \end{array}\right. }$$

### Repair generator functions

The repair generator functions in Table 2 may return two kinds of results. It either suggests: i) sub-nodes (arguments) of complex expressions that need to be explored recursively, or ii) if a leaf of an evaluation tree is reached, it suggests repair actions such as *add* (add a new property to a model element or add a model element to a collection), *mod* (modify a model element property), or *del* (delete a model element property, or remove a model element from a collection). Depending on the quantifier type and the expected result, one or more elements must be deleted or added from this model element property. The condition of the quantifier provides one or more repair sequences and/or alternatives that are generated recursively depending on the type of expressions the condition consists of.

Consider, for example, the universal quantifier of rule #9 in Table 2. If this quantifier is expected to be *true* ($exp=t$), then every element in the quantifier collection must satisfy the quantifier condition. If the quantifier evaluates to *false*, then we have two alternatives for every element $re_{bi}$ in the collection where the condition evaluates to *false*: i) We could delete this element from the collection, or ii) we could repair (modify) this element. This is implied by $\langle *,\{\langle del,\, A \, b_{i}\rangle ,b_{i}\}\rangle$, which denotes an alternative between the deletion of the element $b_{i}$ in collection *A* or the repair of the condition $b_{i}$. The first alternative is a simple repair action (Algorithm 2, Lines 12-13). The second alternative is a more complex condition that can be repaired only by exploring its arguments recursively (Algorithm 2, Lines 10-11).

Note that the alternative applies the repair of a single, offending element of the quantifier only. Naturally, all violating elements need to be repaired $\langle +,\{\bigcup _{i=1}^{|A|}\{...|re_{bi} =f\}\}$, which explains the sequence $+$ of repairs needed across the union of all elements of the collection (*A*) where the condition is *false* ($re_{bi}=f$). If the universal quantifier is expected to evaluate to *false*, then the repair changes significantly as the negation of an universal quantifier is equivalent to an existential quantifier with a negated condition (see rule #10 in Table 2). Table 2 describes multiple sets of repair nodes/arguments (an entire sub-tree) for the quantifier whereas the algorithm expects a single node. The actual implementation breaks up the quantifiers into multiple parts that separate collections from conditions. However, doing so makes the table less readable and hence we opted to keep operations intact in the table.

### Repairs for collections and non-Boolean values

In this paper, we also extended previous work [42] by generating repairs for additional expressions, presented in Table 2 (rules #12-#17). The collection expressions presented in rules #12-#14 are Boolean operations, similar to all rules above (#1-#11). Rule #15, however, presents the expression *size* that returns a non-Boolean value, similarly to the *subString* and *concat* expression in rules #16 and #17. Note that for the collection Boolean expressions, the repair generation follows a similar pattern of the universal and existential quantifiers.

In Table 2, rule #12, we have the *isEmpty* expression. As this is a Boolean expression, two expected (*exp*) values are possible, either *true* (*t*) or *false* (*f*). If $exp = t$, then collection *A* should be repaired by removing all its elements. This would generate a sequence of repairs denoted by $\langle +,\{\bigcup _{i=1}^{|A|} \{\langle +,(del,A, a_{i})\rangle \}\}\rangle$, which would iterate over all elements in collection *A*, and delete all its elements. However, if $exp = f$, then *A* is already empty, so adding any value to it (*add*, *A*, ?) would make it consistent. In this case, we can only propose an abstract repair for the developer (thus $v = ?$) because our approach does not handle the generation of concrete repairs as in [25]. We argue, however, that by providing the type of repair to the developer as well as the model element, our approach provides guidance for fixing the inconsistency. Also, there is the possibility that *A* in the *isEmpty* expression is a collection generated from a sub-expression, *e.g.*, self.operations->select(o|o.name = ’play’)->isEmpty(). In this case, the *isEmpty* expression could also be repaired by repairing the sub-expression that generated *A*. Thus, the last two options on rule #12 in Table 2 are called if *A* is an expression ($A \in \epsilon$). If the expected result is *true*, all elements in the collection must be repaired in accordance with the sub-expression (thus, a sequence of repair actions $+$). For doing this, there are two alternatives, deleting the element ($del, A, a_{ai}$) or repairing the element based on the sub-expression. Considering the second alternative, if the *select* expression selects all operations from *Streamer* that have their name equals to “play,” it would result in a non-empty collection as two operations have this name (see Fig. 1a). To repair the sub-expression, we should change the name of the operations to something different than it currently is. Thus, the expected value from the sub-expression should be the opposite of the actual value ($mod, a_{i}, \lnot re_{ai}$). This leads to all elements in the collection generated from the sub-expression being repaired until none of them satisfies the *select* sub-expression. If the expected value of the *isEmpty* expression is *false*, then the *select* expression is returning an empty collection. Thus, the operations of class *Streamer* should be changed so that at least one element satisfies the sub-expression name equals “play” (thus, an alternative list of repair actions $*$). The alternative to repair sub-expressions such as this is also present in the other collection expressions described in the following. For simplifying the explanation, however, we only consider the main expression when explaining the repair generation.

Furthermore, for *includes* (rule #13) the repairs would be either to *add* or *del* elements from collection *A* depending on the expected result ($add,\ if\ exp\ = t\ or\ del,\ if\ exp = f$). For *includesAll* (rule #14), however, element *B* is a collection as well, so we can *add* or *del* elements from both collections *AandB*. If the expected result is *true*, then we have two alternatives. For each element from *B* not present in *A* $\langle +,\{\bigcup _{i=1}^{|B|}\}\rangle$, we can either add it into *A* ($add, A, b_{i}$) or remove it from *B* ($del, B, b_{i}$). If the expected value is *false*, there are also two alternatives. The first is to remove from *A* all elements also present in collection *B* $\langle +,\{\bigcup _{i=1}^{|B|}\{(del, A, b_{i})\rangle \}\}$. The second alternative is an abstract repair, as we can add a new element into *B* which is not present in *A* (*add*, *B*, ?).

Our approach also supports the non-Boolean expression *size*. To generate repairs for this case, we have to consider the operator comparing the size of the collection (*a*) with the expected result. Hence, we have four possible operators ($=,\ne ,>,<$) with two different conditions for the equal operator. In Table 2, rule #15, when the operator is “=”, we have to check if the expected value is greater or lower than the result of the size expression $re_{a}$. For instance, if the expected value is greater, then we have to add elements to that collection until we reach a greater size than the expected value (thus we want $re_{a} > exp$). Furthermore, we will have a number *x* of repairs, where *x* will be the difference between the expected value and the actual size of the collection.^5^ If $exp < re_{a}$, the repairs would remove elements from the collection until its size is equal to the expected value. For the other operators, we follow a similar logic; however, the expected result does not affect the repairs generated as the operators different, greater than, and less than are enough information for generating the repairs. As stated before, however, we cannot determine which elements are added or removed from the collection; thus, repairs, in this case, are always abstract. However, our approach provides the correct number of elements to the developer, guiding the developer through fixing the inconsistency.

Lastly, Table 2 presents the *subString* and *concat* expressions (rules #16 and #17) that are related to String operations. These two expressions also have to consider the operator of the parent node, *e.g.*, the “$=$” in $a.subString(0,1)=x$ represents the operator of the parent node, which is an equals expression (rule #6). For *subString*, if the parent operator is “$=$”, we can repair it by modifying the string *a* to an unknown value that satisfies the parent condition (*mod*, *a*, ?). If the parent operator is “$\ne$”, the repair action required is to change the value of *a* into any value that is different than *a* ($mod, a, \ne a$).

For *concat*, the repair could be either to modify the value of the element *a* or the argument *x*. The principle is similar to the *subString* repairs. If the operator from the parent is “$=$”, then either *a* or *x* must be modified to a value that would validate to *true* when compared to a string. In this case, we have two abstract repair alternatives (*mod*, *a*, ?) or (*mod*, *x*, ?). If the parent operator is “$\ne$”, then the value of *a* or the value of *x* must be changed to something different than their currently values. Thus, we have the two repair alternatives ($mod, a, \ne a$) or ($mod, x, \ne x$). These are also abstract as the value could be any string with the exception of their current values. The generation of concrete values, however, is not the focus of our work. However, by providing these abstract repairs to the developer, we give the guidance needed to start repairing these non-Boolean expressions.

### Raw and flatten repair trees

**Fig. 5:**
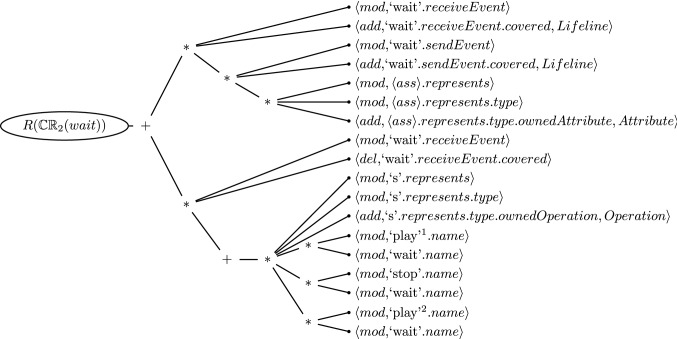
Repair Tree for the Inconsistency of Consistency Rule 2 evaluated on the Message *wait*

When generating a repair tree for CR 2 evaluated on message *wait*, the result would be the one shown in Fig. 5. Recall that this consistency rule had a top-level conjunction and both arguments of that conjunction were wrong: The message direction of *wait* was wrong, and there was no *wait* operation in the *Streamer* class. Hence, the top node of the repair tree reflects the repair of the conjunction where both sides need repairing (hence the $+$). The next level reveals how each argument of the conjunction can be repaired.

The first argument is about the repair of the existential quantifier of the left-hand argument of the conjunction. Looking at Table 2, we find that i) we could modify the property call that identifies over which the quantifier iterates (*self.receiveEvent*), ii) we could add an element to the property *self.receiveEvent.covered* that satisfies the condition of the quantifier, or iii) we could repair some elements accessed by the condition of the quantifier itself. The first two options are simple model changes, and the third option is more complex, comprising yet another existential quantifier. It in turn reveals two alternatives to repair the source of the quantifier (one modify and one addition). The third alternative of this quantifier again suggests to repair its condition, which is the third existential quantifier in the consistency rule. This last quantifier results in yet again three alternatives for repairing the source of the quantifier, namely two modifications and one addition of an element. Finally, the implication provides no repair alternatives because the source of the last existential quantifier does not have any elements. This can be seen in the equality relation on the left-hand side of the implication that compares if the attribute *a* is not null.

Thus far we discussed the repair of the first argument but recall that both arguments of the conjunction were *false*. The second branch below the top level node in the repair tree (Fig. 5) suggests repair actions for the violated universal quantifier. The first alternative is again a repair action to modify the first property call in this chain of property calls (*self.receiveEvent*). The second alternative is the deletion of an element from the property *self.receiveEvent.covered*, and the third alternative is the repairing of the elements accessed by the condition of the quantifier. All violated evaluations of the condition must be repaired and therefore a sequence repair node is added to the alternatives of the universal quantifier repairs. The condition is evaluated only once (only one element is in the source of the quantifier) and is represented by the existential quantifier that iterates over a collection of operations. Therefore, the first alternatives are again the property calls of the source. In this case, there are two modifications (*l.represents*, and *l.represents.type*), and one addition. The alternatives for repairing the existential quantifier are the evaluations of the condition. In the evaluation tree, we saw that the quantifier condition was evaluated three times (the right-hand side of Fig. 3), one time for each operation in the class *Streamer*. The three alternatives consist of two repair actions caused by the equality relation of the quantifiers condition that compares the name of the message (*self.name*) with the name of the operation (*o.name*). There, the first alternative is the modification of the operation name (*play*$^{1}$, *stop*, *play*$^{2}$) and the second alternative is the modification of the message name.

**Fig. 6:**
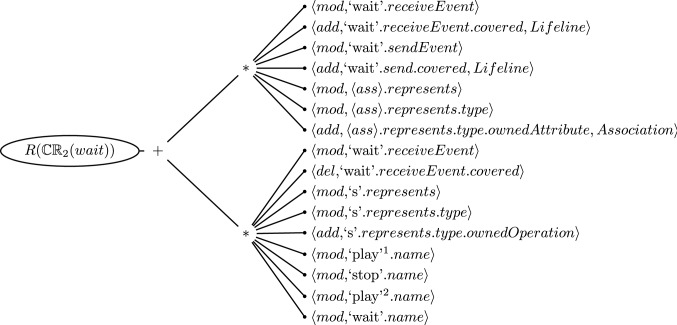
Flattened Repair Tree for the Inconsistency of Consistency Rule 2 evaluated on the Message *wait*

**Fig. 7:**
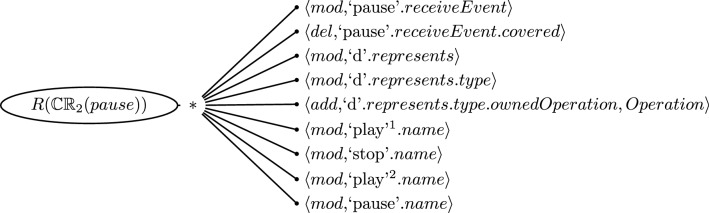
Flattened Repair Tree for the Inconsistency of Consistency Rule 2 evaluated on the Message *pause*

We consider this repair tree (Fig. 5) to be *raw* as it shows all possible repair actions for fixing the inconsistency, even redundancies such as $\langle mod,`wait'.name$ which appears three times. These redundancies can be flattened by eliminating nodes with only one branch (the sequence repair node caused by the conjunction and universal quantifier) and by eliminating cascading nodes (the alternatives nodes). The flattening is a straightforward process that follows the following rules: (i) Nodes with only one branch (regardless of the type) are merged to the parent node, (ii) an alternative node with only alternative children can be merged, (iii) a sequence repair node with only sequence repair node as a child is merged, and, finally, (iv) duplication can be eliminated. Figure 6 depicts the semantically equivalent, flattened repair tree that consists of two branches beneath the sequence node. Hence, possible repair alternatives are combinations of the two branches.

In total, this repair tree has 13 repair actions and the hierarchy of sequences and alternatives allows for 63 repair alternatives with one or two repair actions each. This example also suggests that the repair tree is likely a more suitable means of expressing repairs because it is cognitively easier to select one repair from each sub-branch of the repair tree, *i.e.*, two selections among 9 or less repair actions, rather than enumerating and selecting from the significantly larger number of repair alternatives, *i.e.*, one selection among 63 alternatives. A usability study would be needed to confirm this which is the focus of future work.

It is important to note that we often find that the cause of the inconsistency is small enough that the repair tree is limited to a single branch of sequences or alternatives. Figure 7 depicts such a case for the inconsistent CR 2 evaluated on Message *pause* where only one of the two branches is violated. In this case, the number of repair actions of the repair tree is in fact that same as the number of repair alternatives. Here, the repair tree does not provide any cognitive advantages.

### Highlighting and filtering repairs based on ownership

Although the flattening mechanism simplifies the selection of repairs for the developer, there is still an opportunity for improving the way repair actions are represented in the tree. For this, we explore the ownership property of model elements. Thus, we propose to use the ownership of an element for highlighting repair actions if they suggest modifying elements in the owners’ context. For instance, if the context is a class diagram, we can use artifact-based ownership to highlight repair actions that affect model elements inside that class diagram. This strategy can also be applied for user-based ownership, in which the highlighted repair actions represent those where the model elements being affected are owned by the current user of the tool. Furthermore, the repair actions not highlighted can be used for retrieving information about other owners, *i.e.*, owners of other model elements. This information will clarify which users require to perform each repair action in collaboration for fixing an inconsistency.

Algorithm 3 demonstrates this highlighting mechanism. It starts by iterating over the children of a repair node (Line 2). If a child is a repair node as well, a recursive call on the “highlightActions” function is performed (Line 4). Otherwise, the child is a repair action, thus the “checkOwnership” function from Line 11 is called on Line 5, passing the action and the owner as an argument. The repair action contains the model element that is affected by the repair. In Line 13, it is checked if the list of owners of this element contains the owner passed as an argument. Back at Line 5, if the result from the Boolean function on Line 13 is *true*, then that repair action is highlighted.

**Fig. 8:**
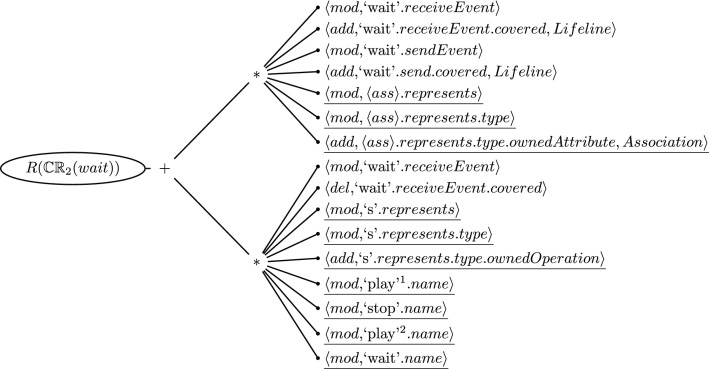
Repair tree highlighted for the inconsistency of CR 2 evaluated on the Message *wait*. Repair actions underlined are those where model elements being affected are “owned” by the class diagram


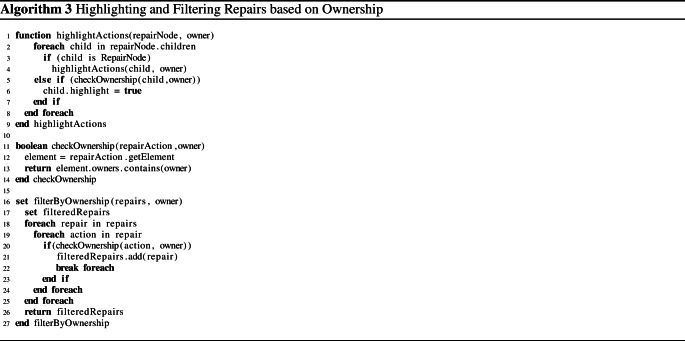


We extend this approach by using the highlighting for generating and filtering a set of possible repairs from the repair tree. More specifically, in addition to the repair tree structure, our approach also provides a list with all possible repairs generated based on the repair tree. Recall that the leaf nodes of the repair tree are repair actions, and a repair is a set of repair actions that fix an inconsistency (Definition 6). This set of repairs is generated based on the repair nodes, and repair actions of the repair tree. If we consider Fig. 6, for example, a generated set of repairs would contain all possible combinations between the alternatives fixing the first part of CR 2 (alternative node at the top) and the alternatives for fixing the second part of CR 2 (alternative node at the bottom). The set combines both options because of the sequence node (+) on the root, which means that there is an conjunction in the repair tree. As the top alternative node has seven alternatives, and the bottom node has nine, the total number of possible repairs would be 63. Consider that this is only an example, and more complex rules may lead to bigger repair trees with multiple conjunctions, thus, multiple sequence nodes.

**Fig. 9:**
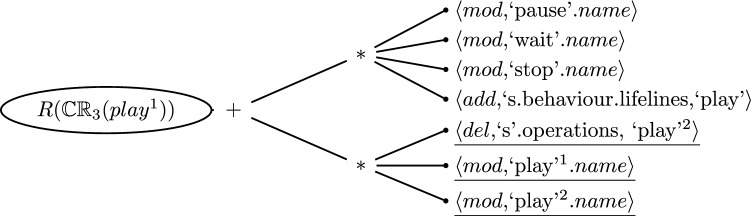
Repair tree highlighted for the inconsistency of CR 3 evaluated on the Message *play*. Repair actions underlined are those where model elements being affected are “owned” by the class diagram

However, when considering the highlighting based on artifact-ownership in the context of a class diagram, we can reduce the number of repairs. Figure 8 shows which repair actions are affecting model elements related to the class diagram (highlighted with underline formatting). Although the tree structure and size are the same, the highlighting identifies those repair actions that can be performed only considering the class diagram. In this context, there are three actions at the first alternative node, and seven in the second. Hence, we could use this information for reducing the set of repairs (previously containing a total of 63 possibilities) to 21 possibilities. However, this might not work in a different tree, as illustrated in Fig. 9, because all the highlighted actions are on the same alternative node. Hence, among the highlighted repair actions, there is no possible combination for fixing both parts of the conjunction. Thus, our filter cannot maintain only the repair actions highlighted, but rather, filter out repairs (set of repair actions) that do not contain at least one repair action highlighted. In this sense, the number of possible repairs for CR 2 on message *wait* would reduce from 63 to 55. However, in the case of CR 3 for operation *play*, the number would remain the same.

Algorithm 3 demonstrates the filtering mechanism process in Lines 16-27. The function “filterByOwnership” receives a list of possible repairs from the repair tree, alongside the owner. Then, it iterates over the repairs (Line 18), and their repair actions (Line 19). For each action, the “checkOwnership” function from line 11 is called on line 20. If the function returns a *true* value, *i.e.*, the owner owns a model element affected by that action, the repair is added to the set of filtered repairs (Line 21) and the iteration ends for that repair (Line 22). This means that if only one repair action from that repair affects a model element owned by that owner, such repair will be included in the list. If none of the repair actions in the repair affect model elements owned, then this repair will not be included in the filtered set. This set of filtered repairs is returned to the user (Line 26).

By considering the ownership of model elements, our approach can also provide developers a highlighted repair tree and a filtered set of repairs. This may aid the developer when deciding which repair actions to execute. As we intend to provide a generic approach, the developer can decide if they want this highlighting and filtering mechanism to be used. Furthermore, the ownership can be based on different contexts, while in this paper we discussed the artifact-based and user-based context, which might allow developers to use different strategies for assigning and using the ownership of the model elements.

### Support for different models types

**Fig. 10:**
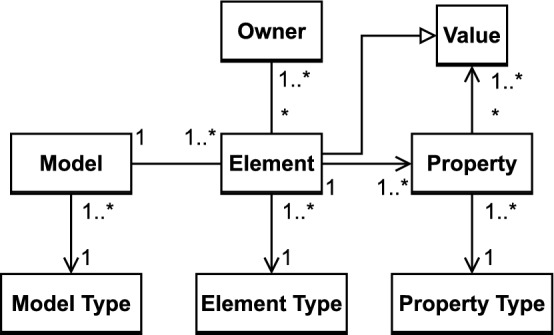
UML Model for representing model elements from different domains

To give support for consistency checking and repair generation for different types of models, we implemented our approach into a supporting tool, namely DesignSpace-IR.$^{1}$ DesignSpace-IR is part of the DesignSpace project$^{2}$, which supports collaborative work of engineers from different domains. Thus, our tool gives support to UML models similarly to the Model/Analyzer tool [42]. However, we extend this support as DesignSpace-IR provides the transformation of models into a common structure, based on the UML model presented in Fig. 10. Hence, a model is of a model type, *e.g.*, UML, source code, and consists of model elements. These elements have a type and properties. Also, elements have owners. As this is a generic model, these owners may have different types depending on the context. In this paper, we consider two types of owners: artifacts, *e.g.*, class diagram (artifact) owns classes (model elements), and users, *e.g.*, developer owns a class and its operations. Continuing, the properties of an element have a type and values. Elements are a sub-class of values, as a model element can also be a value, *e.g.*, class *Streamer* is a value of the property *associationEnd* in the association between classes *User* and *Streamer* (Fig. 1a).

We developed a standalone plugin that transforms models from specific types into the generic type specified in Fig. 10. Following this strategy allows us to extend the support to any type of model only by implementing new plugins that transform models using the same generic structure. After the transformation, our tool loads the transformed model and applies the consistency checking mechanism. For instance, if we consider the class diagram in Fig. 1a, this model is of the type UML. *Streamer* is a model element of the type *class* with properties, such as the operations *play* and *stop*. These operations are also model elements of the type *operation*. The operation *play*, for instance, has a property called *name* with the value equals to *play*. If consider the artifact-based ownership, *Streamer* is owned by the class diagram. Thus, the transformation keeps all original elements, properties, and values from the original model, preventing data-loss that could impact the consistency checker [49].

By transforming the models using the common structure presented in Fig. 10, we can use the same strategy for the consistency checker and the repair generation. Furthermore, the consistency rule syntax for the consistency checking does not change independently of the model type. However, because different model types have different properties, the consistency rules have to be designed specifically for that domain. For instance, consistency rules designed for UML will probably not work for source code as the UML meta-model and source code structure are not the same. For instance, in UML a model element representing a class can have “associations” as a property. In Java, an element representing a class would represent these associations as “fields.” Thus, a rule designed for UML to access property *associations* from a class has to change for Java to access the *fields* of the class.

**Table 3:** Evaluation results for each model

UML models	#mes	#ev (#${\mathbb {I}}$)	#$\overline{ras}$	#*ras*
VOD	71	76 (3)	3	26
ATM	204	253 (12)	114	115
Microwave Oven	292	156 (12)	11	92
MVC	385	350 (57)	40	298
eBullition	448	320 (58)	46	533
Curriculum Planner	621	568 (56)	106	598
Inventory Sales	1064	809 (77)	251	1673
Dice	1100	756 (109)	85	831
Tele-operated Robot	1257	736 (31)	22	138
Course Registration	1261	802 (113)	634	2611
Home Appliance Contr.	1464	923 (106)	382	2190
Vacation and Sick-Leave	1466	1127(117)	504	1716
IOCF05aT12	1540	994(185)	1028	2035
DESI	1979	1779 (276)	385	1266
iTalks	2048	2062 (196)	397	4679
Build. Management	2093	1259 (278)	273	2474
Biter Robocup	2636	2205 (389)	215	1673
Calendarium	2796	2397 (378)	1017	2540
UMLLCAF03aT1	3009	1547(138)	468	546
XVNPI	5857	3351 (529)	1991	5772
dSpace	8771	5660 (1005)	784	4717
oodt	9853	10835 (2013)	1895	8594
Word Pad	11419	7003 (1485)	320	7385
V Insurance Network FC	15619	10772 (3309)	679	18264
*Java Systems*				
chess	12055	6038 (1226)	8653	3175
gantt	51169	38978 (8402)	45318	22080
iTrust	63098	37846 (7223)	45670	18383
jHotDraw	92820	47698 (11900)	74296	30456

#mes - number of model elements; #ev—number of evaluations; #${\mathbb {I}}$—number of inconsistencies;

$\#\overline{ras}$—number of abstract repair actions; #*ras*—number of concrete repair actions

### Concrete repair actions

The repair algorithm discussed earlier generates abstract repair actions. In some cases, however, our approach is also able to compute concrete repair actions because the evaluation tree contains concrete values which we can use. For example, if we want to rename the message *wait*, there are an infinite number of strings available as concrete repair values. However, only two strings would lead to consistency, namely *play* and *stop*, and these two strings can be found in the evaluation tree. How to compute concrete values would exceed the scope of this paper and may be found in [25, 26].

## Evaluation

We evaluated our approach in terms of usability and scalability. We also argue the correctness and minimalism of the approach. Furthermore, we evaluated the impact of highlighting and filtering repairs based on the model elements being modified. In the following sections, we describe our evaluation protocol, results and analysis, and threats to validity.

### Evaluation design

To evaluate our approach, we defined different sets of consistency rules, one for each type of model. More specifically, we defined 17 rules for UML (see Appendix A) and 14 for Java code (see Appendix B). All these rules were written using our consistency-language syntax, which is a simplified version of OCL, *e.g.*, instead of having “OCLAsType”, our language has “asType” expression. Furthermore, the models used were gathered over the course of many years and most are proprietary, *e.g.*, Inventory Sales is owned by IBM Rational and was shipped with IBM Rational Rose as a sample UML model. The evaluations were carried out using our consistency checking tool (see Sect. 4.11). The specifications for the execution environment are an Intel Core i7-7700 CPU @3.6GHz with 16GB (8GB available for the tool) RAM and Windows 10 x64-based. The evaluation results, UML models, Java source code, and the CRs are available at our online repository [30].

Table 3 shows the models used in our evaluation as well as the number of model elements (#mes). The third column of Table 3 shows how many evaluations (#ev) were conducted in each model for checking the consistency. This column also shows how many inconsistencies (#${\mathbb {I}}$) were found, *i.e.*, how many evaluations returned *false*. For instance, the *ATM* UML model has 204 model elements, and it was evaluated 253 times from where 12 inconsistencies were found. For fixing these inconsistencies, 114 abstract ($\overline{ras}$) and 115 concrete (*ras*) repair actions were generated, 229 in total. These results show that for each inconsistency in the ATM model, an average of around 19 repair actions is being provided to the developer. In the following sections, we discuss the results.

It is important to mention that the goal of our evaluation is to collect data regarding the practicality of our approach. Thus, the data collected aim to show how the inconsistency repair behaves in practice, rather than in theory. The main reason for this is that our approach should be applied in practice during modeling/designing. Hence, we argue that our results are true for practical cases, rather than universally valid (for theoretical cases).

### Usability results

**Fig. 11:**
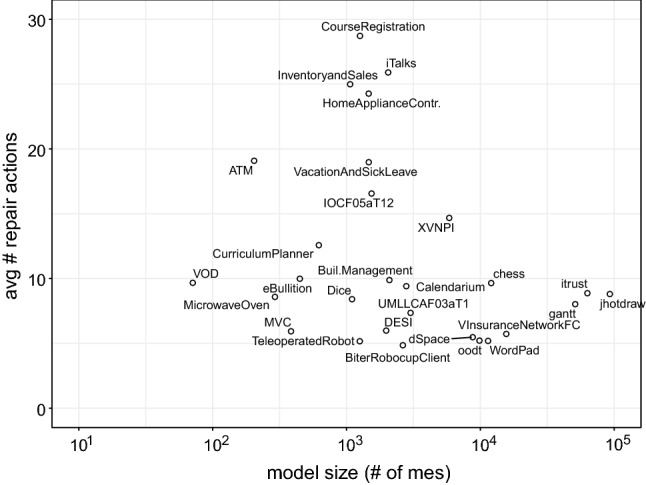
Average number of repair actions by model size

**Fig. 12:**
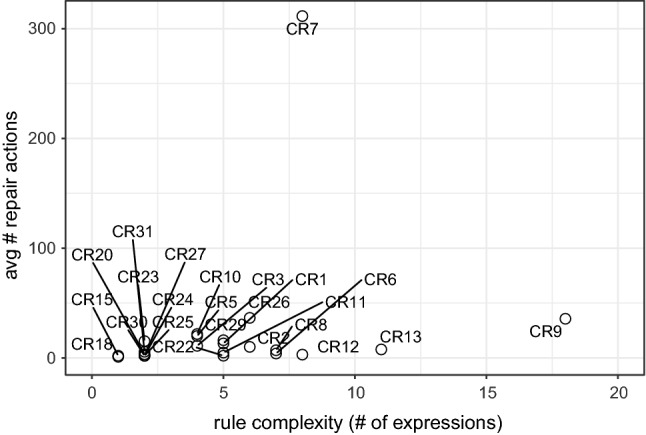
Average number of repair actions by rule complexity (number of expressions)

Considering the results in Table 3, we notice that the bigger models do not always generate a greater number of inconsistencies. This can be seen, for instance, when comparing models *oodt* and *Word Pad*. The former has less #mes but has more inconsistencies. As we used the same set of consistency rules for all models, this indicates that some models are more consistent than others. Thus, analyzing the total number of inconsistencies and repair actions is not enough for achieving conclusions about the usability of our approach. However, considering the average of these numbers provides us interesting data. Figure 11, for example, shows that the average repair tree sizes (number of repair actions) ranged from five to nine per model considering the model size (#*mes*). Most of these repair trees were flat in containing single “alternatives” and “sequences” only. In this figure, we can notice that the average number of repair actions is not influenced by the model size. This can be seen as repair trees for bigger models, such as *jhotdraw* and *iTrust*, averaged less than ten repair actions. In contrast, repair trees for models *CourseRegistration*, *iTalks* and *InventorySales* averaged between 25 and 30 repair actions. This result is important for understanding the usability of our approach, as the results show that, on average, the number of repair actions in a repair tree is reasonable for a developer to deal with.

Figure 12 compares the average size of the repair trees per consistency rule, considering the complexity of the rule (number of expressions). As can be seen, most rules generate an average number of repair actions ranging from 1 to 50. We can also see that the number of the expressions within the rule does not impact the average number of repair actions. This can be seen as CR7 (see appendix A) is the rule with more repair actions on average, while not being one of the most complex rules. In contrast to CR9, the rule with more expressions is within the average size range of most other rules. Thus, the number of model elements within a model and the complexity of the rule do not always affect the size of the repair tree. This happens because depending on the consistency rule, the number of elements evaluated, *i.e.*, accessed by the rule, varies. This is more explicit if we analyze the structure of the rules to understand what impacts the average number of repair actions in a repair tree. If we consider CR7, we can see that its context is *Package*, and the rule checks the list within a package. Thus, the rule is checking every model element inside the list of *packagedElement* for evaluating the consistency. The first element in a UML model is a package, which contains a list of packages with packaged elements inside. Hence, a large number of model elements are being evaluated, and if found to be inconsistent, need to be modified. Thus, the results shown in Fig. 12 represent that this particular rule has a large number of repair actions due to a large number of model elements being evaluated. Based on this analysis, while considering the conclusions mentioned earlier, we can conclude that:
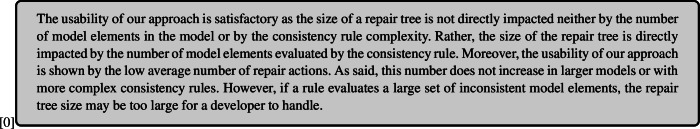


### Scalability results

**Fig. 13:**
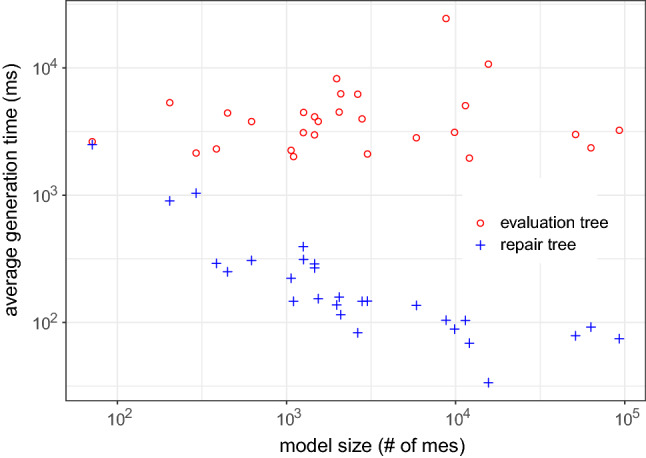
Average time for generating evaluation and repair trees by model size

**Fig. 14:**
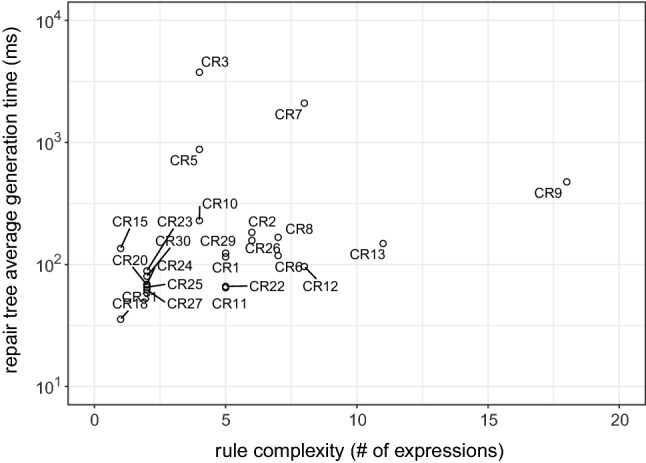
Average time for generating repair tree by rule complexity

To evaluate the scalability of our approach, Fig. 13 shows the average time, in milliseconds (ms), for generating the evaluation and repair tree for each model. In this figure, we can see that the generation of the evaluation tree is always higher than the repair tree. While the evaluation ranges from more than $10^3$ to less than $10^5$ ms on average, the repair tree generation ranges from below 10 up to $10^3$ ms on average. This represents that, on average, our approach takes 4.69 seconds for generating an evaluation tree and 0.3 seconds for generating a repair tree considering the evaluated models. The reason for this difference is that for generating the evaluation tree, all model elements in the context of the rule are evaluated. For the repair tree, however, only inconsistent model elements are considered. As the number of inconsistent model elements will never be greater than the total number of model elements, these results were expected. We can also see that the average generation time remained small and stable regardless of the model size. This result has shown that our approach is scalable as it is applicable in models of different sizes as the trees’ generation time is not impacted by the number of model elements being checked.

Considering the rule complexity, the size of the evaluation tree is related to the time for computing it. Thus, as we discussed in the usability results, consistency rules with iterative expressions (*forAll*, *select*, *exist*) tend to evaluate more model elements. However, the number of model elements evaluated also depends on the context of the rule. For instance, in a UML model, there are more *packagedElements* than classes, *e.g.*, CR7 presented the repair trees with the bigger sizes. Hence, the time needed for the generation of the evaluation tree is not the complexity of the rule (number of sub-expressions), rather the number of model elements evaluated by the rule. This is also evidenced by the average repair generation time which is presented in Fig. 14. Most of the repair trees were generated, on less than $10^2$ms on average, except for CR9, CR5, CR7, and CR3, which are between $10^2$ and $10^4$ms. As argued, these results show that the complexity of the rule by itself does not affect the generation time in a significant way, as the growth is linear. The variable that always impacts both the generation time and the size of the trees is the number of model elements accessed by the rule. To clarify this, we can consider CR3 (appendix A), the CR with the greater average time for generating repair trees, and CR12, which had one of the smaller average times. CR12 is a more complex rule than CR3 as it contains more sub-expressions; however, CR3 evaluates more model elements because its context and types of expressions consider all associations in the UML model.

We highlight these results as they go in a different direction from what theoretical results show [5]. In theory, the size of the model should have a direct impact on the time for evaluating the elements. This happens because the more elements a model has, the more evaluations are performed, and the more inconsistencies could be found, leading to more repairs to be computed. However, this is directly related to the rule definition. For instance, a consistency rule to compare all elements’ names with all other elements’ names would generate, in the worst case, $\#me^2$ evaluations. Thus, bigger models would take more time and resources to evaluate than smaller models. However, such rules are may not be used in practice. As we mentioned, our goal when elaborating our approach is to use it in practice during the designing of models. This claim is supported by analyzing which consistency rules are found in other approaches [22, 26, 33, 36, 44]. We still plan, however, to conduct further research in the regard of how well-defined consistency rules must be for the approach to work properly. Hence, by analyzing our findings we conclude that, regarding scalability:
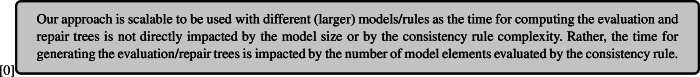


### Correctness and minimalism results

The correctness of our approach essentially hinges on the correctness of the cause computation. Nentwich *et al.* [33] demonstrated that their approach must be complete (no missing repair actions). As was discussed, our approach essentially extends on [33] by removing non-minimal repair actions by considering the cause of inconsistency. Hence, our approach always computes a subset of the repair actions computed by [33]. Recall that the causes of inconsistencies are almost always a subset of the model elements involved in the computation of inconsistencies [44]. In the subset provided by our approach, however, repairs that do not repair the cause directly are not considered (side effects). So, we argue that the correctness and minimalism of our approach rely on the correctness and minimalism of the mechanism for removing non-minimal repairs.

First, let us discuss if our approach is correct. To compute the cause of inconsistency, we compare the expected result of an expression with the evaluated result. The evaluated result is simply observed during the evaluation of a consistency rule. In this sense, an inconsistency cannot be caused if the expected result is equal to the evaluated result. Hence, all expressions where the expected result equals the evaluated result can be ignored safely during repair computation. This implies that our approach is conservative, because the expressions ignored are not related to the cause. Thus, repairs generated from these expressions would not repair the cause of a given inconsistency. Therefore, ignoring these expressions not related to the cause does not impact the correctness of the approach.

Considering the minimalism, we have to discuss if our approach produces minimal repair alternatives and if it is an optimal result with regard to the cause. For this, we have to consider the possibility of an expression where the expected result is different from the evaluated result, while this expression does not cause a given inconsistency. This case is possible whenever an expression is irrelevant to a parent’s expression. Consider the disjunction $a\vee b$. If both *a* and *b* evaluate to *false*, then both cause the disjunction to be *false*, *i.e.*, an inconsistency. However, if only *a* evaluates to *false* and *b* does not, then the expected result of *a* differs from the evaluated result, as *a* is expected to evaluate to *true*. In this case, we have the situation where the expected/evaluated results of *a* differ. This difference, however, is irrelevant to the parent’s expression $a\vee b$ whose expected result still equals the evaluated result. Algorithm 2 avoids this problem through the top-down recursive exploration of expressions, which does not explore an expression’s argument unless the expression’s expected result differs from the evaluated result. Consequently, there cannot be any repair actions for expressions where the expected and evaluated results differ, while the parent’s expressions do not.

Based on the aforementioned, we can define the minimalism of our approach based on the difference of our work and Nentwich et al. [33], as illustrated in Fig. 2. While the approach of Nentwich et al. [33] provides a set of all possible repair actions (${\mathbb {A}}$), our approach provides a subset of repair actions (${\mathbb {B}}$) which only contains repair actions that repair the cause of an inconsistency $\langle {\mathbb {B}} \subset {\mathbb {A}}\rangle$. However, as we do not consider side effects that may or may not be repaired later, repair actions present in ${\mathbb {B}}$ only represent the starting point for the developer to start fixing all inconsistencies in the model (one at a time). Thus, it is possible that some repairs present in the subset ${\mathbb {B}}$ are more complete (fix multiple inconsistencies) than others. Hence, we argue that our approach must be minimal with regard to the cause of an inconsistency.
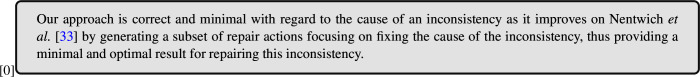


### Evaluating the ownership-based filter

The last analysis of the results is related to the repair filtering considering two different types of ownership, namely user-based and artifact-based. The goal of this evaluation is to check if the filtering based on the ownership presents similar results independently of the type of ownership applied (either artifact-based or user-based).

For user-based ownership, we aim at collecting evidence that our filter brings benefits for a variety of scenarios. For this, we simulated four different scenarios for collecting data: (i) ownership of all model elements (100%); (ii) ownership of 75% of the model elements; (iii) ownership of 50% of the model elements; and (iv) ownership of 25% of the model elements. Thus, we configured all four scenarios for each model by randomly selecting the model elements to be owned until the total number represented the respective percentage for each scenario, *i.e.*, 100, 75, 50, and 25. We used the same models described in Table 3. After assigning this ownership, we generated the repairs and filtered them. The reason for performing this strategy for the evaluation is to mitigate the threat of not being able to simulate a real user scenario. In this configuration, we can analyze our approach results in a variety of possibilities and see if the results differentiate based on the scenarios.

**Fig. 15:**
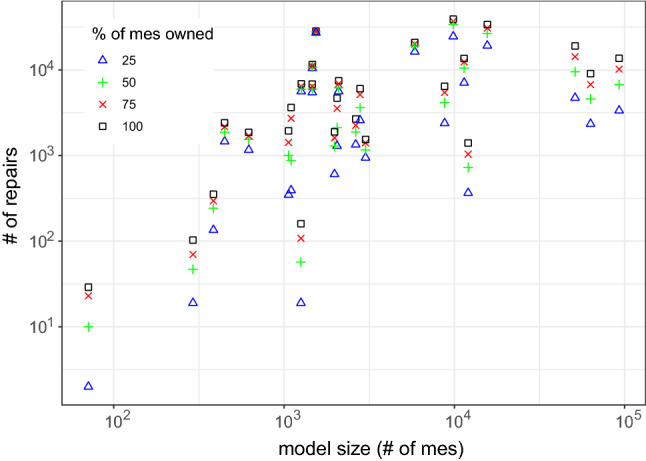
Repairs per Model Filtered by User–Ownership

Figure 15 shows the total number of repairs per model based on the different user–ownership scenarios. The number of repairs generated ranged from less than 10 up to almost $10^5$. As shown, for most models, reducing the number of elements owned also reduced the number of repairs generated. The percentage of repairs filtered was around the same percentage of model elements owned. Thus, when 75% of the elements were owned, the repairs remaining after the filter represented 75% from the total number of repairs without filtering. The reason for this is related to the usability results mentioned earlier. As we mentioned, the size of the repair tree is impacted by the number of inconsistent model elements. The repair tree also impacts the set of generated repairs. Thus, by reducing the number of owned elements, the number repair actions highlighted also reduces. This impacts the number of generated repairs, as repairs with no-highlighted repair actions are filtered from the set.

There were some cases where the user-based ownership filter did not reduce the number of repairs following this pattern. This happened when the repair trees generated presented a high amount of alternatives modifying a single model element. Thus, as this single element might have been owned in all four scenarios, the number of repairs generated from this tree would remain the same. This indicates that the user-based filter presents better results *when the repair tree contains repair actions modifying different model elements*.

**Fig. 16:**
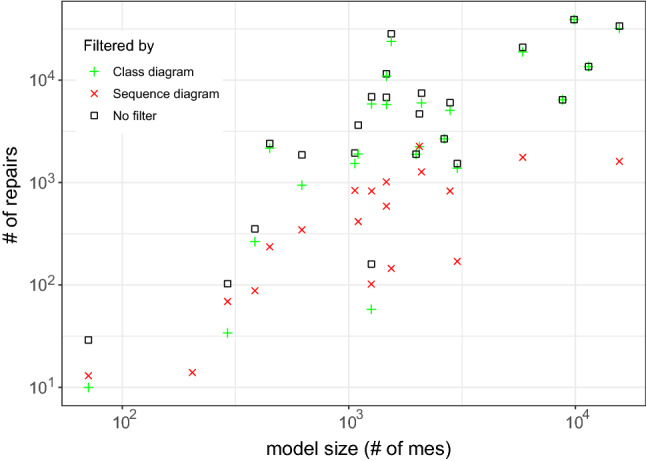
Repairs per Model Filtered by UML Diagram Type

For evaluating the artifact-based filter we separated the UML and Java source code, as these use different types of artifacts. For the UML models, we considered the ownership of different diagram types, namely class and sequence. Thus, a filter for class diagrams would filter out repairs modifying model elements that were not part of a class diagram. Figure 16 illustrates the results of each filter, as well as no filtered results, per each UML model. For most of the models, by filtering out repairs of different diagrams, the number of repairs reduced. Only in five cases the number of repairs with no filter and with the class diagram filter was the same. This happened because some models did not contain inconsistencies outside their class diagrams, and thus, all repairs generated were fixing this type of diagram only. Thus, our filter presented better results *when applied in a repair tree modifying model elements from different diagram types.*

**Fig. 17:**
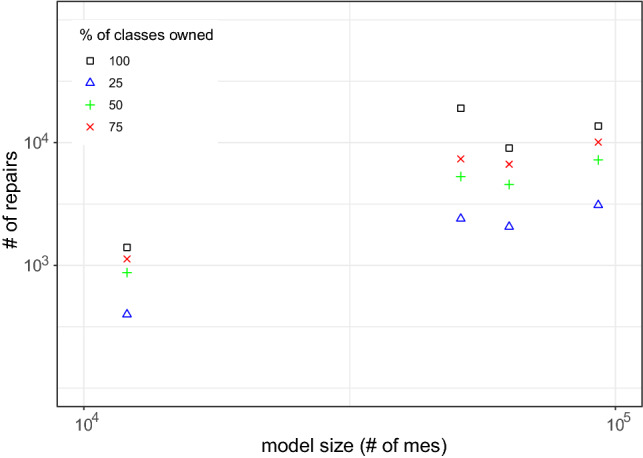
Repairs per Model Filtered by Ownership of Java Classes

For the Java source code, we filtered the repairs based on the Java classes. Thus, if a class was owned, all the elements within this class were also owned, *e.g.*, fields and methods. However, as the number of classes varied among the models, before collecting the data we assigned the ownership of the classes similar to the user scenarios. More specifically, we randomly assigned the classes of each model in four different scenarios: (i) All classes were owned; (ii) 75% of the classes were owned; (iii) 50% of the classes were owned; and (iv) 25% of the classes were owned. Figure 17 shows the results of the four scenarios for Java. By reducing the number of classes owned, the number of repairs remaining after the filter also reduced. Similarly to the user-based ownership, when 75% of the classes were owned, the number of repairs remaining after the filter was applied was around 75% of the total. The same pattern happened for 50 and 25%. Thus, in this case, *our filter presented similar results in all scenarios, not being impacted by the model size or consistency rule*.

**Fig. 18:**
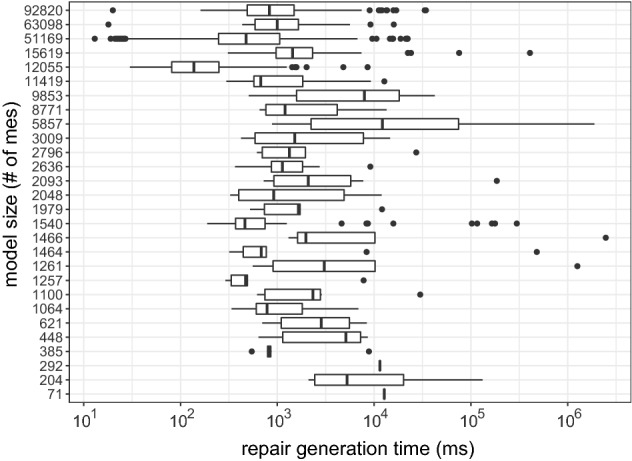
Repairs Generation Time per Model

For collecting evidence about the scalability of our repair generation, we collected the time in milliseconds required for generating the repairs from a repair tree for each model. This result is shown in Fig. 18. We can see in this figure that the average time for generating the repairs is not always impacted by the model size. We can also see that the generation time took on average between 0.1 seconds ($10^2$ ms) and 100 seconds ($10^5$ ms), with only a few outliers below and above this range. This resulted in an average of 8.3 seconds for generating the repairs for all models. Hence, we understand that the repair generation and filtering are scalable as the model size does not impact the time required for generating the repairs. Also, the time required is within a satisfactory range.

Based on the results discussed, we can conclude that: 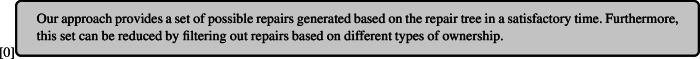


## Threats to validity

This section discusses internal, external, and conclusion threats to validity and how we mitigated them based on [53].

### Internal validity

An internal threat is related to the selection of the models used for the evaluation. In this threat, a set of only small or large models could result in too many or too few inconsistencies found, thus also impacting the repair generation. For mitigating this threat, we used 24 UML and four Java systems with their sizes ranging from 71 to 92,820 model elements. This resulted in a range from 3 to 11,000 inconsistencies per model, aggregating in a total amount of 39,683 inconsistencies, 1417.25 on average. These numbers show that the models used are varied enough for supporting our findings.

Another threat is the set of consistency rules used as the evaluation and repair tree sizes may be impacted by the rules. To mitigate this threat, we defined a varied set of rules with different sizes, expression types, and evaluating different contexts. The results regarding the number of evaluations per model support our claim that this threat was mitigated. The evaluations number ranged from 76 to 47,698, resulting in a total of 187,300, 6,689 on average. These numbers show that more than 50% of the model elements were evaluated for each model. Thus, we argue that the consistency rules were varied as they were not only checking the consistency of a small set of model elements per model.

### External validity

An external threat is related to the generalization of our results to other model domains. In this evaluation, we used UML models and Java source code, so we only have evidence to support our approach in these domains. Our approach, however, can be extended to other domains. For this, we provided and discussed a generic structure (Fig. 10) that can be used from transforming models from different domains. After this conversion, the consistency of these models can be checked by defining the rules based on that domain.

### Conclusion validity

A conclusion threat is related to the scenarios considered for the evaluation of the user-based ownership filter. As we did not use a real scenario with real users, the results may be impacted. For mitigating this problem, we randomly assigned the model elements considering four different scenarios. These different scenarios configured as 25, 50, 75, and 100% of the model elements were owned. These different ownership scenarios provide us with a variety that can be used for collecting data about the usability and scalability of the filter. However, we acknowledge that user-based ownership is still not adopted in the UML modeling community yet, as it is considered still a challenge even in cross-domain collaborative tools [49]. As our approach does not focus only on UML, however, we believe that giving support for this type of ownership is important. In source code, for instance, user-based ownership is being applied by different version control systems, *e.g.*, Git^6^, as well as in project management tools, *e.g.*, Jira.^7^

We also considered the artifact-based filter for achieving our conclusions. For the artifact-based filter of Java, we also defined four different scenarios. Our goal was collecting data considering this variety of ownership of the Java classes. Our results show that for both cases, the filter follows a pattern as the number of repairs reduces based on the number of model elements owned. As mentioned before, the main reason for using the four possible scenarios with different percentage of ownership is to obtain results considering this variety of possibilities. By analyzing these results we could check if our filtering mechanism was behaving differently according to the scenario. As the results showed, this was not the case as the filtering kept a pattern among all scenarios.

## Related work

The approach presented in this work relies on analyzing consistency rules that are expressed in first order predicate logic [24, 50]. However, a key aspect is that our approach detects the cause of an inconsistency based on the structure of a consistency rule and the behavior during its evaluation. The novelty of our approach is the combination of the structure of the consistency rule and its evaluation behavior while most existing approaches focus on the structure only (or a few on the behavior only). Despite this, our approach is similar to most existing approaches in using a language for consistency rules based on predicate logic [26, 32, 54]. Thus, our approach can still be applicable to all of them in principle. In this section, we discuss these pieces of work, presenting their similarities and differences compared to our approach. We structure this discussion based on the different topics that are relevant to our work.

### Cause of inconsistencies

A pre-requisite for correct and meaningful repairs is the calculation of the real cause of an inconsistency. In the SAT community, there is such an equivalent: a Minimal Unsatisfiable Set (MUS) [28] which is the minimal set of unsatisfied clauses that cause a UNSAT, which is analogous to inconsistent. In this case, a design model and its constraints can be transformed into an SAT model [10], followed by applying (High-level) Unions of Minimal Unsatisfiable Sets ((H)UMUS) [34]. However, this might lead to problems because the models and constraints have to be transformed into SAT, then the (H)UMUS calculation is performed. This is a problem due to the computational needs being expensive and not necessarily incremental. Thus, we argue that it is beneficial to identify causes directly in design models reducing these computational needs. Furthermore, Czarnecki and Pietroszek [10] use OCL to define well-formedness rules for the verification of feature-based model templates which are analyzed by a SAT solver. While not directly related to the aforementioned problem, the ability to translate constraints requires detailed understanding of constraint semantic which is very relevant in this work. König and Diskin [23] proposed an algorithm for consistency checking on interrelated models to reduce cost of inconsistency detection caused by model-merging. They achieve this reduction by performing early localization based on the specified formalization of the inter-relation on an arbitrary number of typed models. However, they dot not propose repairs for these inconsistencies.

Nentwich et al. [32] provide an incremental approach (xLinkit) to evaluate the consistency of arbitrary XML documents. However, their approach is conservative and it may suggests non-minimal and even incorrect repairs, for instance, if the consistency rule is partially violated only, *i.e.*, if not all parts of a consistency rule evaluation that access model element properties contribute to the cause of an inconsistency. This happens because their approach uses the structure of a constraint for generating and filtering repairs for inconsistencies. Thus, it does not use the actual cause of the inconsistency. This is a problem because the cause is usually different for every inconsistency. Nonetheless, we will see that our approach builds on their work. Table 2 is clearly based on their principles. Like them, we do not consider side effects on other constraints.

### Repair generation

Hegedus et al. [19] present an approach that is based on graph transformations which generates quick fixes for Domain-Specific Modeling Languages (DSMLs). They use a graphical notation to express the model and the constraint, which is different comparing to our strategy. Based on the DSMLs, the approach creates quick fixes to resolve an inconsistency using Constraint Satisfaction Problem over models. The quick fixes contain as many actions as needed to resolve a given inconsistency. Document management systems (DAGs) check the consistency of interrelated documents that are processed by a team of authors. For example, Scheffczyk et al. [46] use s-DAGs [47] to represent the documents and the consistency rules. Repairs for inconsistencies are derived from the s-DAG representation and not from the documents. Heuristics are used to eliminate unnecessary repairs. These heuristics may be useful in their field, but they are not necessarily useful for model-based software development, because each software project has different requirements. Thus, no generic heuristics can be derived and applicable for all software projects. Furthermore, the generation process of repairs is independent of the inconsistency detection process which requires additional computation as opposed to our approach, where the repair generation is built directly on top of the consistency detection mechanism.

Xiong et al. [54] present an approach that combines the detection of errors and provides actions to repair them on UML models. They use their own language to define the consistency relations. This language, called *Beanbag*, has an OCL-like syntax and provides a fixing semantic for elements that are changed. However, when writing consistency relations, the developer also has to specify how this relation has to be fixed when it is violated, a manual and error prone activity without guarantee for completeness or correctness. Dam and Winikoff [11] analyzed and developed an approach on how OCL constraints, based on their internal structure, can be violated or resolved, respectively. They distinguish five different actions that can be taken to achieve a violation or resolution. Abstract repair plans are generated at compile time, *i.e.*, the set of OCL constraints is statically defined in the tool, and these abstract actions are instantiated if the constraint is violated by the model. The repair plans that resolve the inconsistency are ranked and provided to the user who decides which plan to execute. The repair plans can also be modified or executed partially. Their approach is designed exclusively for OCL and a proof is given that this approach is correct and complete regarding single OCL operations. However, in contrast to [33], this approach considers all inconsistencies at once which is both a scalability problem as recognized by the authors and not necessarily in the spirit of tolerating inconsistencies [4].

Straeten and D’Hondt [48] use a knowledge base (expressed in description logic) as well as the query and rule language nRQL to generate repairs for inconsistent models. The inconsistencies are detected by nRQL queries where the variables of these queries are bound to model elements. The resolutions are represented as nRQL rules that consist of statements that add or remove data from the model to resolve the inconsistency. This approach also considers all inconsistencies at once and generates a set of repair actions that transform the model from an inconsistent state to a fully consistent one, if a solution exists. As their approach must transform the model and the inconsistency rules into description logic, it has no incremental characteristic, *i.e.*, the operation is similar to batch-based approaches that are time consuming. Moreover, the same limitations as for Dam and Winikoff [11] apply. An incremental approach for detecting and repairing inconsistencies is also presented in [12, 13]. It uses various languages for the definition of consistency rules, like C# or Java and is extended to OCL for the definition of dynamic constraints (consistency rules). It does not need any annotations or modifications of existing languages to check the consistency of UML models. Based on this approach, Egyed [13], Egyed et al. [14] present how to repair inconsistencies in models and how the generated choices are evaluated. However, their approach is overly conservative and generates repairs for all model elements accessed by the evaluation of an inconsistency, while often only a subset thereof causes the inconsistency. Nonetheless, our work borrows extensively from these approaches. Particularly, in that the repair tree is computed from an evaluation tree which is a refinement of their approach.

### Repair planning

Almeida da Silva et al. [3] developed a Prolog-based approach that generates repair plans for inconsistencies. These repair plans consist of actions in Praxis notation that are needed to resolve as many inconsistencies as possible causing as few new inconsistencies as possible. As there exists an infinite number of ways to resolve inconsistencies, this approach has a configurable exploration level that reduces the number of repairs with the danger of not resolving the inconsistencies. Favoring minimal changes with maximal consistency is a heuristic that may be useful in certain cases but does not explore the breadth of the repair alternatives available, and it thus has limitations in applicability.

Puissant et al. [41] propose to use automated planning for resolving design model inconsistencies while aiming at a fast computation of repairs without assessing the relevance of the repair plans. With a similar goal, the work of Ohrndorf et al. [35, 36] traces unfinished modifications to identify the cause of inconsistencies and propose possible repairs. Our approach differs from these by checking for inconsistencies and proposing repairs incrementally. Allaki et al. [2] focus on repairing inconsistencies in collaborative modeling scenarios. They organize and analyze the decision making concerning the inconsistency fixing, thus aiding the repair process. Their approach handles repairs from a management perspective, focusing more on the decision-making on how to handle the repairs. Our approach, however, focus on generating repairs based on the source of the inconsistencies, as well as providing a tree structure to help the selection of the generated repairs.

### Non-repair-based approaches

There are approaches that emphasize on instrumentality and optimizations but are not necessarily amendable to repairs. Xu et al. [55] described how consistency rules can be optimized for the re-evaluation. They use a run-time observation of the consistency rule evaluation to filter out parts of the evaluation that do not contribute immediately to the overall evaluation result. The filtering is not, like in this paper, optimized to detect the cause of an inconsistency but to optimize the memory consumption and re-evaluation time for the pervasive context where the resources are limited. While their approach is able to detect inconsistencies, it is not able to compute the cause of an inconsistency. Nonetheless, we borrow from the idea of trying to understand the evaluation to optimize a process, although we do so for a different purpose.

Blanc et al. [7] introduced an incremental approach that is based on the model changes that can be made. In their approach, a consistency rule will be re-evaluated only if a certain change in the model affects the result of the consistency rule. They pointed out that the performance of re-validating a consistency rule depends on the complexity of the consistency rule condition, but little is known about the overall scalability. Nonetheless, understanding whether a change impacts a consistency rule is related to understanding repairs though their paper does not explore this aspect. Jongeling [21] proposed a methodology that helps developers to maintain consistency. This is done by identifying inconsistencies during the development and maintenance of the system. The work also reports how to mitigate these inconsistencies, without relying on repairs.

### Studies on concrete repairs and side effects

Two limitations of our approach are not generating concrete repairs and not dealing with side effects generated from repairing an inconsistency. Kretschmer et al. [25] propose the generation of concrete repairs based on abstract repairs generated. Khelladi et al. [26], Kretschmer et al. [22] dealt with change propagation of models considering repairs and their side effects. However, these approaches focus on how to transform abstract repairs into concrete ones, and how the side effects of a repair impact other repairs, rather than the generation of repair alternatives, the focus of our approach. Also, our approach supports highlighting and filtering the repairs generated based on the model elements being modified. Another difference from their work is that our tool also supports different types of models besides UML.

Wang et al. [51, 52] present ARepair, an automated tool for repairing Alloy models [20]. Their tool captures desired model properties through the use of AUnit tests, repairing the model so that all AUnit tests pass. Relying on unit tests gives information for repairing inconsistent models automatically, which may be a good option depending on the engineering scenario. In addition, the BeAFix tool [18] also supports automated repair of Alloy models. Their approach, however, does not rely on unit tests, but on assertions on the models, which are used in formal declarative languages. Our approach, however, goes toward a different direction of these approaches, as we do not intend to repair the models automatically. Rather, our approach aims at giving the developer a subset of repair alternatives that is manageable and applicable to its intention.

### Database repairs

Finally, it is important to point out that the repair of design models has conceptual similarity to database repairs when integrity constraints are violated [5]. Thus, database repair techniques have been applied for fixing inconsistencies in UML/OCL conceptual schemas [16]. Rull et al. [45] present a tool (AuRUS) that analyzes UML/OCL conceptual schemas and presents the result of consistency checking of integrity constraints. These integrity constraints describe the reason why a property of the schema was not satisfied, causing an inconsistency. We may also find other tools with the similar goal of checking for problems in UML schemas [8, 9, 16]. All these approaches provide a way of formalizing a UML schema by relying on the constraints defined. They mostly, however, only support specific UML diagrams, such as class diagrams [9].

A more generalizable approach is presented by Oriol et al. [39]. With this approach, given a structural schema with its constraints and an information base, the consistency of such information base can be maintained after the application of some update. Their approach relies on a chase algorithm for computing repairs, a technique applied in database repairs [1]. Their approach was evaluated using UML schemas with constraints defined in OCL$_{FO}$ [15], a fragment of OCL. The limitation of their approach is the need of a schema-like structure with constraints defined in the OCL$_{FO}$, thus restricting its applicability in other types of artifacts as well as using constraints defined with regular OCL. While our approach can be applied with regular OCL, as well as to different types of artifacts such as source code, a similar approach is presented by Krieger et al. [27], called OCLexec, which translates OCL constraints defined in the conceptual schema into an SAT problem. Then, it simulates the operation by generating Java source code that invokes an SAT reasoner. This reasoner provides an information base that is guaranteed to satisfy the OCL constraints defined. This work is also restricted to situations where schemas are present. Furthermore, due to the technologies limitations of using Java, their approach is limited to specific types of repairs, *e.g.*, the approach is not able to deal with deletions. Also, none of these approaches consider the owner of the artifacts being checking for consistency, which we support with the ownership concept. This support can provide a way to perform collaborative repairs among users.

## Conclusion

This paper presented an approach for generating repair trees for fixing model inconsistencies. Our approach achieves this result by focusing on identifying the cause of the inconsistency and generating a repair tree with a subset of repair actions focusing on fixing this cause. We also discussed how we build on past work by including in the approach support for collection and non-Boolean expressions. Furthermore, we implemented a highlighting mechanism based on ownership. With this mechanism, our approach can generate a set of repairs from the repair tree, filtering out repairs modifying model elements not owned by the developer.

The approach was implemented as a standalone tool, and we discussed how we extended this tool to support consistency checking and repair generation for different types of models. We empirically evaluated our approach on a set of 24 UML models and four Java systems. For collecting the data of the evaluation, we used a set of 17 UML consistency rules and 14 Java consistency rules. The data were collected in a context of 39,683 inconsistencies identified in the models. It was shown that the focus on the causes of inconsistencies provides a more manageable repair tree. In terms of usability, the size of the repair trees generated per model ranged from five to nine on average. The time for generating these repair trees averaged around 0.3 seconds, showing the scalability of our approach. We also found that the computation time of the evaluation and repair trees was independent of the model size, which can vary widely. We argue that our approach is correct and minimal with regard to the cause, due to improving on [33] with the difference that we focus on the cause of the inconsistency, thus removing non-minimal repairs from the repair tree. In addition, we evaluated our approach considering an ownership-based filter. The results evidenced that this filter can reduce the number of possible repairs generated from a repair tree based on the number and type of model elements owned by a developer.

For future work, we plan to conduct an evaluation with real users, as well as use additional types of models such as Jira^8^ and 4diac^9^ to extend our evidence concerning the support for different models. We also plan to conduct evaluations considering models from other domains. Furthermore, we plan to mine source-code repositories to obtain user-based ownership information from artifacts. This information may be considered for applying the inconsistency repair approach, for filtering out repairs as well as expanding the collaborative aspect of repairing artifacts. Lastly, we aim at investigating how well-defined a consistency rule must be to have impact in the scalability of our approach.

## A: Consistency rules (CR) for UML models

**CR 1:** Message Action must be defined as an Operation in Receiver’s Class

**CR 2:** The connected Classifier of the Association End should be included in the Namespace of the Association

**CR 3:** AssociationEnds must have unique Names within the Association

**CR 4:** At most one AssociationEnd may be an Aggregation or Composition

**CR 5:** A Class may use Unique Attribute Names

**CR 6:** A Classifier may not belong by Composition to more than one Composite Classifier

**CR 7:** A Package must have a non-empty unique name

**CR 8:** An Interface can only contain Public Operations and no Attributes

**CR 9:** No two Class Operations may have the same Signature

**CR 10:** Operation Parameters must have unique Names

**CR 11:** The Type of Operation Parameters must be included in the Namespace of the Operation Owner

**CR 12:** The Parent must be included in the Namespace of the Generalizable Element

**CR 13:** Statechart Action must be defined as an Operation in the Owner’s Class

**CR 14:** An Operation has at most one return Parameter

**CR 15:** Every lifeline has to have a corresponding Class

**CR 16:** Every transition has to have a corresponding message

**CR 17:** A lifeline without messages must not have a corresponding transition

## B: Consistency rules (CR) for Java

**CR 18:** A method has to be owned by a Class

**CR 19:** An interface must be implemented by a class

**CR 20:** A class must implement all methods from its interfaces

**CR 21:** A class can only have one super class

**CR 22:** A class should have methods and fields

**CR 23:** A field name must be unique within the class

**CR 24:** A field name should not be longer than 20 characters

**CR 25:** A field should be accessed by at least one method

**CR 26:** A method name within a class can only be the same if the parameters are different

**CR 27:** A method name should not be longer than 20 characters

**CR 28:** A method must be called at least once

**CR 29:** A method should have more than 0 and less than 11 statements

**CR 30:** A method should have less than 11 parameters

**CR 31:** A method should have parameters with unique names

## Footnotes

1 IR stands for **I**nconsistency **R**epair.

2 https://isse.jku.at/designspace.

3 From now on, we use the term *evaluation* to refer to a consistency rule being evaluated in a model element.

4 Note that our algorithm ignores shortcut evaluation. For example, if the first argument of a conjunction is *false*, then the second argument is still evaluated which is unnecessary for determining the evaluation result but useful for obtaining a more complete picture of the cause.

5 For simplifying this repair in Table 2, we assumed that our iteration would start with $i = re_{a}$ and finish when $i = exp+1$.

6 https://git-scm.com/.

7 https://www.atlassian.com/software/jira.

8 https://www.atlassian.com/software/jira

9 https://www.eclipse.org/4diac/
